# Climatic modulation of surface acidification rates through summertime wind forcing in the Southern Ocean

**DOI:** 10.1038/s41467-018-05443-7

**Published:** 2018-08-13

**Authors:** Liang Xue, Wei-Jun Cai, Taro Takahashi, Libao Gao, Rik Wanninkhof, Meng Wei, Kuiping Li, Lin Feng, Weidong Yu

**Affiliations:** 1 First Institute of Oceanography, State Oceanic Administration, Qingdao, 266061 China; 2 Laboratory for Regional Oceanography and Numerical Modeling, Qingdao National Laboratory for Marine Science and Technology, Qingdao, 266237 China; 3 School of Marine Science and Policy, University of Delaware, Newark, DE 19716 USA; 4 Lamont–Doherty Earth Observatory of Columbia University, Palisades, NY 10964 USA; 5 NOAA Atlantic Oceanographic and Meteorological Laboratory, Miami, FL 33149 USA

## Abstract

While the effects of the Southern Annular Mode (SAM), a dominant climate variability mode in the Southern Ocean, on ocean acidification have been examined using models, no consensus has been reached. Using observational data from south of Tasmania, we show that during a period with positive SAM trends, surface water pH and aragonite saturation state at 60°–55° S (Antarctic Zone) decrease in austral summer at rates faster than those predicted from atmospheric CO_2_ increase alone, whereas an opposite pattern is observed at 50°–45° S (Subantarctic Zone). Together with other processes, the enhanced acidification at 60°–55° S may be attributed to increased westerly winds that bring in more “acidified” waters from the higher latitudes via enhanced meridional Ekman transport and from the subsurface via increased vertical mixing. Our observations support climatic modulation of ocean acidification superimposed on the effect of increasing atmospheric CO_2_.

## Abstract (web summary)

The Southern Annular Mode is a dominant climate variability mode in the Southern Ocean. Using observational data, the authors show a strong climatic modulation of ocean acidification via Southern Annular Mode -related winds.

## Introduction

The Southern Ocean has naturally low pH and saturation states of calcium carbonate (CaCO_3_) due to cold temperatures and upwelling of CO_2_-enriched deep waters, and it is vulnerable to ocean acidification (OA) caused by increasing atmospheric CO_2_ levels^1–4^. Surface waters of the Southern Ocean are predicted to become undersaturated with respect to aragonite (a more soluble form of CaCO_3_ relative to calcite) as early as year 2030 if sea surface CO_2_ increases in concert with atmospheric CO_2_ (ref. ^3^). OA, defined as declining pH or CaCO_3_ saturation states over decades or longer timescales^5^, affects many marine organisms and especially fragile Southern Ocean ecosystems^6–8^. Although global OA is due primarily to increasing atmospheric CO_2_ by fossil fuel combustion and land use changes since the Industrial Revolution^2,9^, it may be enhanced by other processes such as upwelling, eutrophication, sea ice melt, and anomalous ocean circulation^10–17^. Such rapid acidification challenges the evolutionary adaptation capacity of organisms^18^. Therefore, understanding the processes or factors that modulate OA is important for projecting impacts on marine organisms and ecosystems.

**Fig. 1:**
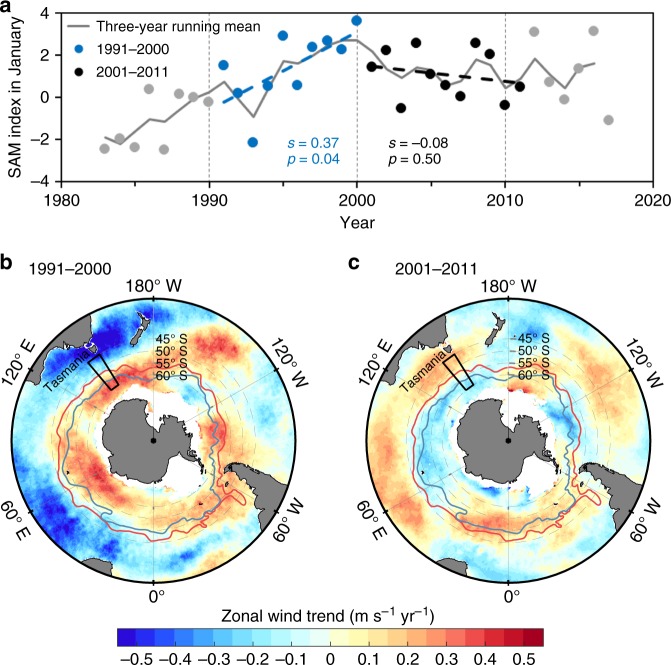
SAM index and change rates of zonal wind speed in the Southern Ocean. **a** SAM index in January 1983–2017 calculated by Marshall^20^. **b**, **c** Change rates of zonal wind speed in January 1991–2000 and January 2001–2011. In **a**, change rates of the SAM index (slope values) during the periods 1991–2000 (blue) and 2001–2011 (black) were determined using an ordinary least squares linear regression; slopes (*s*) and *p*-values of the regression analyses are also shown (differentiated with blue and black colors for the two periods). The gray line shows the weighted three-year running mean of the SAM index, which splits the data into two decades. In **b**, **c**, the red and blue lines show the mean positions of the subantarctic front (SAF) and the polar front (PF)^58^, respectively; the black rectangle delineates the study area south of Tasmania. Change rates of zonal wind speeds, which are based on the CCMP wind product, were calculated using an ordinary least squares linear regression in each grid (0.25° × 0.25°)

Climatically, the Southern Ocean is sensitive, particularly during austral summer, to the Southern Annular Mode (SAM) that is the dominant mode of climate variability in the extratropical Southern Hemisphere^19,20^. This mode is quantified by the SAM index as the difference in normalized mean sea level pressure between 40° and 65° S (ref. ^20^). In January, there was a positive SAM trend towards a high-index particularly since the 1980s, but this trend changed around 2000: the following decade exhibited decreased or no significant SAM trends (Fig. 1a). A positive SAM trend is associated with increasing westerly winds at high-latitudes (south of 55° S, Fig. 1b) resulting in increased equatorward Ekman transport and vertical mixing. Thus, “acidified” waters with lower pH and aragonite saturation state (Ω_arag_) from the south and from deeper depths are likely to be transported to the surface further north. Therefore, enhanced surface OA in excess of the effect of increasing atmospheric CO_2_ may be expected at high-latitudes during a period with positive SAM trends. Here we define enhanced OA as evidenced by declining rates of pH or Ω_arag_ that are faster than rates predicted from increasing atmospheric CO_2_ alone.

However, due partly to lack of observational data, previous studies on the effects of SAM on OA in the Southern Ocean use models which yielded different and even opposite conclusions^18,21–23^. Therefore, it is necessary and important to further investigate the mechanistic role of the SAM on sea surface carbonate chemistry and OA. Also, it is important to ascertain whether OA responds to the SAM differently for different latitudinal zones as was shown for circulation and biology^24^, since the SAM measures a seesaw of atmospheric mass between the high-latitudes and mid-latitudes of the Southern Hemisphere^20^.

Given that the region south of Tasmania is perhaps the only region where there is continuous observational CO_2_ data since 1991 (Supplementary Fig. 1), we use observations from this area spanning two decades during 1991–2011, with contrasting SAM trends before and after 2000 (Fig. 1a and Supplementary Figs. 2–3) and show how changing wind patterns related to the SAM affect the rate of surface OA. We find that the SAM appears to have significant modulating effects on OA rates over different latitudinal zones. To account for the SAM modulation of OA rates, we examine mechanisms associated with wind-driven meridional Ekman transport and vertical mixing during austral summer when the upper ocean layers are stratified. Our work helps improve understanding of the mechanisms of OA in the Southern Ocean, thus providing observational constraints for the improvements of prediction models for ocean uptake of atmospheric CO_2_ and impacts on the marine ecosystem.

## Results

### Changes of carbonate chemistry with time

**Fig. 2:**
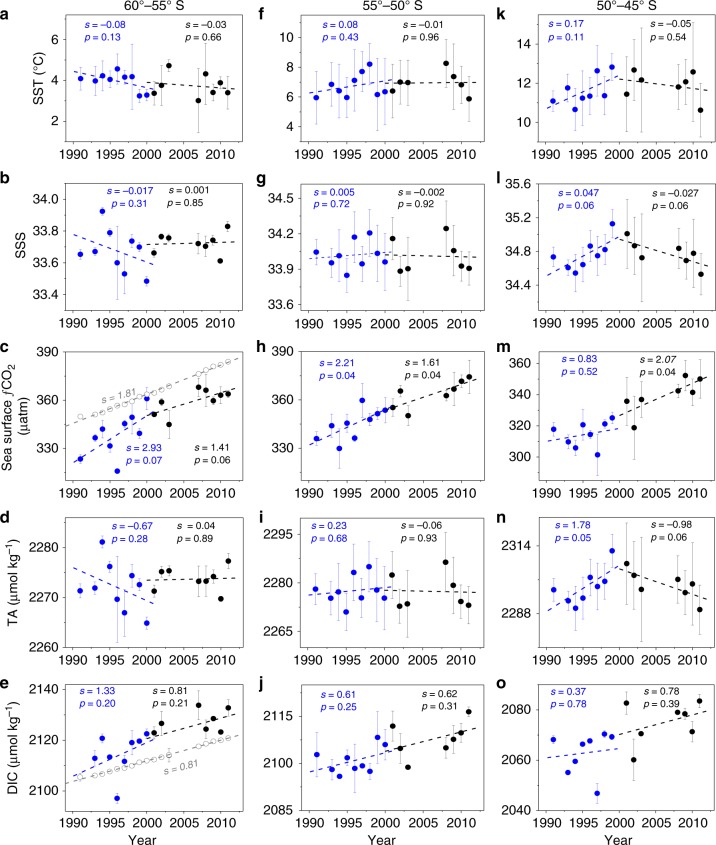
Temporal variability in sea surface temperature, salinity, and carbonate parameters in January in three latitudinal bands. **a**–**e** Sea surface temperature (**a**, SST), salinity (**b**, SSS), sea surface CO_2_ fugacity (**c**, *f*CO_2_), estimated total alkalinity (**d**, TA) and calculated dissolved inorganic carbon (**e**, DIC) at 60°–55° S. **f**–**j** Show the same parameters but at 55°–50° S; **k**–**o** also show the same parameters but at 50°–45° S (see 'Methods'). The vertical bars show one standard deviation, which reflects the spatial variability within each latitudinal band. Linear regression analyses were performed for the periods 1991–2000 (blue) and 2001–2011 (black). Slopes (*s*) and *p*-values of the regression analyses are also shown (differentiated with blue and black colors for the two periods). A trend of *p*-value < 0.1 is regarded as statistically significant (90% confidence interval) due to the small sample numbers (<10). Also, the atmospheric CO_2_ data (shown as *f*CO_2_) observed at the GCO (Cape Grim, Tasmania) atmospheric CO_2_ measurement station (ftp://aftp.cmdl.noaa.gov/data/trace_gases/co2/flask/) and the DIC values computed due solely to the atmospheric CO_2_ increase (see 'Methods') are indicated with open gray circles in Fig. 2c, e

Using observed sea surface CO_2_ fugacity (*f*CO_2_), temperature (SST) and salinity (SSS) from the Surface Ocean CO_2_ Atlas (SOCAT version 2)^25^, and estimated total alkalinity (TA) from SSS, SST, and latitude (Fig. 2), we calculated dissolved inorganic carbon (DIC), pH, and Ω_arag_ over the two contrasting decades, 1991–2000 and 2001–2011 (see 'Methods'). The estimated values of TA and DIC agree well with measured data ('Methods' and Supplementary Fig. 4), giving high confidence in the calculated pH and Ω_arag_. To achieve a better spatial representation, prior to these calculations, the surface *f*CO_2_, SST, and SSS data were binned and averaged within 0.02° latitudinal bands. Then averages were taken for the 5° latitudinal bands of 60°–55° S (high-latitudes or Antarctic Zone), 55°–50° S (transition zone or Polar Frontal Zone) and 50°–45° S (mid-latitudes or Subantarctic Zone)^26^. Finally, these data were adjusted to January values using the climatological seasonal variations described by Takahashi et al.^27^ (see 'Methods'). While trends in SST, SSS, and TA were often not statistically significant, the relative rate of *f*CO_2_ increase in surface water vs. that in the atmosphere was clear over the three regions and both time periods. A faster *f*CO_2_ increase occurred during the pre-2000 positive SAM trend period in the high-latitude zone (60°–55° S), and a slower (or zero) increase in the mid-latitude zone (50°–45° S) compared to the atmospheric increase (Fig. 2).

**Fig. 3:**
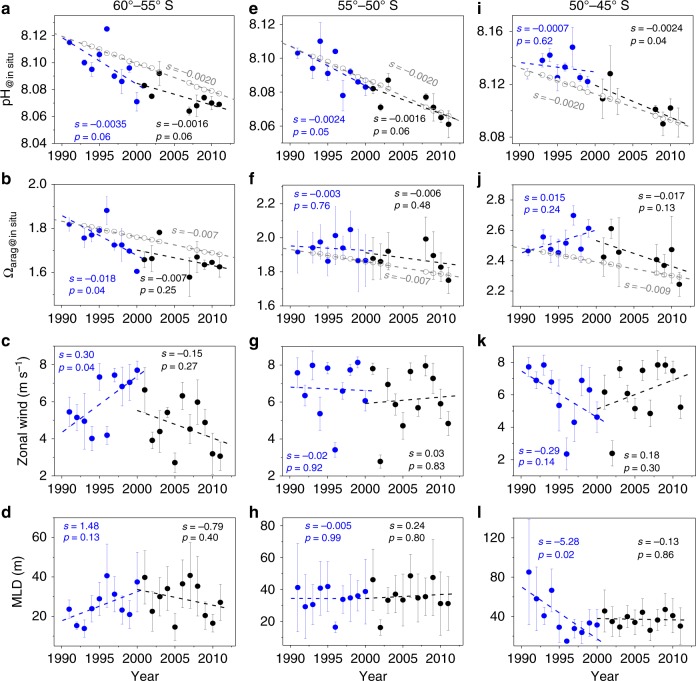
Temporal variability in sea surface pH, Ω_arag_, zonal wind speed and mixed layer depth (MLD) in January in three latitudinal bands. **a**–**d** Sea surface pH at in situ temperature (**a**, pH_@__in_ _situ_), sea surface aragonite saturation state at in situ temperature (**b**, Ω_arag@__in_ _situ_), zonal wind speed (**c**) and mixed layer depth (**d**, MLD) at 60°–55° S. **e**–**h** show the same parameters but at 55°–50° S; **i**–**l** shows the same parameters but at 50°–45° S. The vertical bars show one standard deviation, which reflects the spatial variability within each latitudinal band. Linear regression analyses were performed for the periods 1991–2000 (blue) and 2001–2011 (black). Slopes (*s*), and *p*-values of the regression analyses are also shown (differentiated with blue and black colors for the two periods). Trends of *p*-value < 0.1 are regarded as significant statistically (90% confidence interval) due to the small sample numbers (<10). The open gray circles indicate the values computed due solely to the atmospheric CO_2_ increase shown in Fig. 2c (see 'Methods'). Zonal wind speed and MLD are the mean values within 140°–148° E in the three latitudinal bands, respectively. Note by definition the trend of meridional Ekman transport is the same as that of zonal wind

Figure 3 shows that the rates of pH and Ω_arag_ change (i.e., rate of acidification) correlate with the SAM trends (Fig. 1a). At high-latitudes (60°–55° S), pH at in situ temperature (pH_@__in_ _situ_) decreased faster (0.0035 yr^–1^) during the pre-2000 positive SAM trend than the pH decrease expected from atmospheric CO_2_ increase alone (0.0020 yr^–1^, gray dashed line, Fig. 3a). Correspondingly, Ω_arag_ at the in situ temperature (Ω_arag@__in_ _situ_) decreased at a rate of 0.018 yr^–1^, which is more than twice the rate of 0.007 yr^–1^ due to atmospheric CO_2_ alone (Fig. 3b). During the subsequent decade (2001–2011) when there was no significant SAM trend, pH_@__in_ _situ_ and Ω_arag@__in_ _situ_ decreased at rates in accord with those predicted from atmospheric CO_2_ (Fig. 3a, b).

In contrast, at mid-latitudes (50°–45° S), patterns opposite to those seen in the high-latitude band were observed (Fig. 3). During the decade of positive SAM trend (1991–2000), pH_@__in sit__u_ decreased much slower than would be expected from atmospheric CO_2_, and Ω_arag@__in_ _situ_ even increased, although neither trend was statistically significant. During the subsequent decade (2001–2011) when there was no significant SAM trend, pH_@__in_ _situ_ and Ω_arag@__in_ _situ_ both showed enhanced rates of decrease relative to the atmospheric CO_2_ based prediction (Fig. 3i, j). For the transitional band (55°–50° S), the decrease in surface pH_@__in_ _situ_ during the two SAM periods was not statistically distinguishable from that predicted from atmospheric CO_2_ and there were no significant changes in Ω_arag@__in_ _situ_ (Fig. 3e, f). Overall, acidification rates differ during different SAM-trend periods and within different latitudinal bands, similar to the responses of circulation and biology to SAM^24^, suggesting that the influence of SAM on the acidification rates was likely associated with SAM-sensitive physical and/or biological factors.

### Correlation between wind trend and OA rates

Our results display a consistently negative correlation between pH_@__in_ _situ_ (or Ω_arag@__in_ _situ_) and wind speed, despite varying latitudinal responses of wind speed to the SAM trend (Fig. 3). In the high-latitude 60°–55° S band, wind speed increased significantly during the 1991–2000 positive SAM trend (Fig. 3c), when pH_@__in_ _situ_ and Ω_arag@__in_ _situ_ decreased faster than expected from the atmospheric CO_2_ increase (Fig. 3a, b). During a period with an insignificant change in SAM trends in 2001–2011 when wind speed decreased or did not change significantly, pH_@__in_ _situ_ and Ω_arag@in situ_ declined at rates similar to those expected from the atmospheric CO_2_ increase. In contrast, in the mid-latitude 50°–45° S band, during the period of positive SAM trends when winds decreased (Fig. 3k), pH_@__in_ _situ_ only decreased slightly and Ω_arag@__in_ _situ_ increased somewhat (though not significantly, Fig. 3i, j), whereas during a period with an insignificant change in SAM trends when winds increased (Fig. 3k), pH_@__in_ _situ_ and Ω_arag@__in_ _situ_ decreased evidently (Fig. 3i, j). For the transitional 55°–50° S band, there were no apparent changes in wind speed and, correspondingly, there was no enhanced acidification during the two periods of 1991–2000 and 2001–2011 (Fig. 3e–g).

**Fig. 4:**
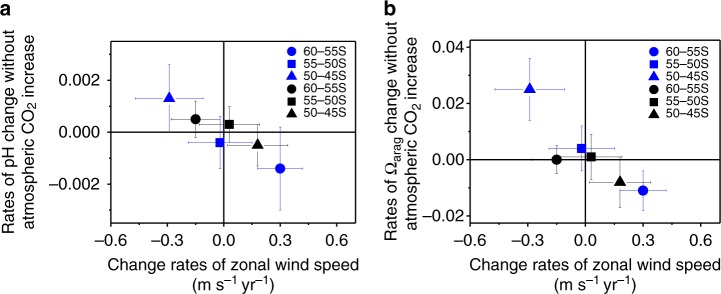
Impacts of SAM associated winds on acidification rates. **a**, **b** rates of surface pH (**a**) and Ω_arag_ change (**b**) without atmospheric CO_2_ increase versus change rates of January zonal wind speed during two periods of 1991–2000 (blue) and 2001–2011 (black) in the three latitudinal bands of 60°–55° S, 55°–50° S and 50°–45° S. Rates of pH and Ω_arag_ change without atmospheric CO_2_ increase are highly negatively correlated with change rates of zonal wind speed with a correlation coefficient of 0.92 and 0.89, respectively. Rates of pH and Ω_arag_ change without atmospheric CO_2_ increase were the observed rates of pH_@__in_ _situ_ and Ω_arag@in situ_ change, subtracting their rates predicted from atmospheric CO_2_ increase alone (see 'Methods'). In this figure, negative change rates of pH or Ω_arag_ denote enhanced acidification compared to that predicted from atmospheric CO_2_ increase alone. The bars show one standard deviation of change rates as shown in Fig. 3

We see more clearly the complex effects of wind on rates of pH and Ω_arag_ change by subtracting their rates of decrease due solely to atmospheric CO_2_ increase from the observed rates of pH and Ω_arag_ change (see 'Methods'). It is clear, after removing the effects of atmospheric CO_2_ increase, that the rates of pH and Ω_arag_ change are negatively correlated with change rates of zonal wind speed over the two periods and the three latitudinal bands (Fig. 4). That is, increasing winds enhance acidification.

### Modulations of Ekman transport and vertical mixing on OA

**Fig. 5:**
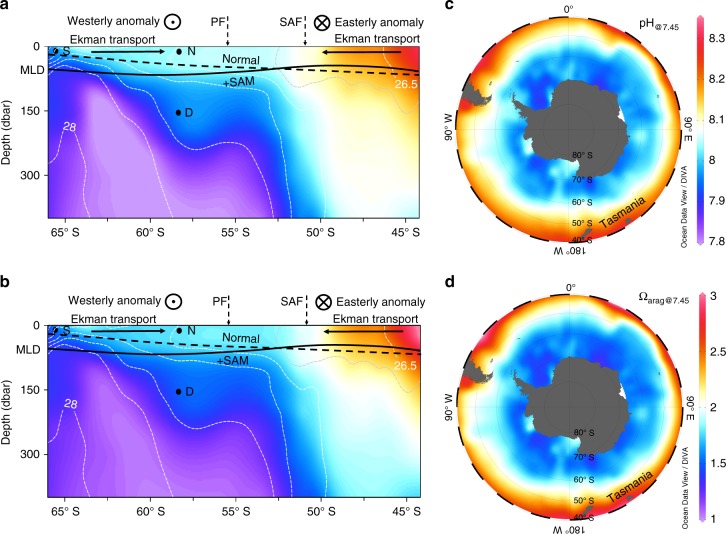
A schematic of Ekman transport and vertical mixing modulation of acidification rates as well as climatological distribution of surface pH_@7.45_ and Ω_arag@7.45_. **a**, **b** Depict changes of wind-driven Ekman transport and mixed layer depth (MLD), and their influences on surface pH_@7.45_ (**a**) and Ω_arag@7.45_ (**b**) in the region south of Tasmania during a positive SAM trend. There will be an anomalous equatorward Ekman transport when westerly winds increase (westerly anomaly), while there will be an anomalous poleward Ekman transport when westerly winds decrease (easterly anomaly). MLD is used for showing the changes in vertical mixing in the upper ocean. Black dashed (normal) and solid (+SAM) lines denote MLD before and during a positive SAM trend, respectively. In **a**, **b**, SAF and PF denote the mean positions of the subantarctic front (SAF) and the polar front (PF)^58^. In the water column, neutral density contours with an interval of 0.25 kg m^−3^ (*γ*^n^, white dashed line), and pH_@7.45_ (**a**) and Ω_arag@7.45_ (**b**) distribution (shaded) observed along Transect SR03 during December 1994–January 1995 are shown. Also in **a**, **b** Points S, N, and D are shown, details about which can be found in Supplementary Table 5. In **c**, **d**, climatological distribution of surface pH_@7.45_ (**c**) and Ω_arag@7.45_ (**d**) in January calculated from the TA and DIC data of Takahashi et al.^9^ is shown. Note **a**, **c** use the same color bar, and **b**, **d** use the same color bar. Figure 5 is plotted using Ocean Data View (odv_4.7.10_w64 version)^59^

Considering the correlation between enhanced pH and Ω_arag_ decreases and zonal wind speed changes (Fig. 4), and the lateral and vertical distributions of pH and Ω_arag_ in the Southern Ocean (Fig. 5), we explore the impacts on surface acidification from lateral transport and vertical mixing, both of which are influenced by wind speeds. Note in this section we used values at the regional mean temperature of 7.45 °C or pH_@7.45_ and Ω_arag@7.45_ to examine the non-thermal influences of pH and Ω_arag_ although temperature influence (thermal influences) on these parameters was relatively minor (see 'Methods'). We considered that, among various drivers listed in Table 1, wind-driven lateral or Ekman transport was one of the important contributors to the trend in pH and Ω_arag_ changes relative to the atmospheric CO_2_ increase. As shown in Fig. 5a, b, surface pH_@7.45_ and Ω_arag@7.45_ in the Southern Ocean decreased poleward. At high-latitudes (60°–55° S) during the positive SAM trend of 1991–2000, the increase in westerly winds (westerly anomaly) enhances equatorward Ekman transport (Supplementary Fig. 5a), causing more waters with low pH_@7.45_ and Ω_arag@7.45_ (“acidified” waters) from further south to be transported to this zone (Fig. 5a, b). This should result in further decreases in pH and Ω_arag_ (enhanced acidification, Fig. 3a, b; Supplementary Fig. 6a, b) in addition to those due to atmospheric CO_2_ increase. In contrast, in the mid-latitude band (50°–45° S), westerly winds decreased (easterly anomaly) during a positive SAM trend, resulting in decrease in equatorward Ekman transport (i.e., anomalous poleward Ekman transport, Fig. 5; Supplementary Fig. 5a) and hence a slight increase in pH_@7.45_ and Ω_arag@7.45_ (Supplementary Fig. 6k, l). This should counteract the acidification by increasing atmospheric CO_2_, thus leading to no clear trends in pH_@__in_ _situ_ and Ω_arag@__in_ _situ_ (suppressed acidification, Fig. 3i, j). Similarly, during the subsequent decade of 2001–2011 changes in pH_@7.45_ and Ω_arag@7.45_ at high-latitudes and mid-latitudes (Fig. 3; Supplementary Fig. 6) were also consistent with those expected by wind-driven Ekman transport (Supplementary Fig. 5b).

**Table 1:** Observed trend change of OA, wind speed, Ekman transport, Ekman pumping velocity, mixed layer depth (MLD), sea surface temperature (SST), salinity (SSS), total alkalinity (TA), dissolved inorganic carbon gains due to air-sea CO_2_ exchange (ΔDIC_a-s_) and chlorophyll *a* in the three latitudinal bands of 60°–55° S, 55°–50° S, and 50°–45° S during the 1991–2000 positive SAM period

	60°–55° S Antarctic Zone	55°–50° S Polar Frontal Zone	50°–45° S Subantarctic Zone	Note
Enhanced OA	+	nc	**−**	Fig. 3
Wind speed	+	nc	**−**	Fig. 3
Ekman Transport	+	nc	**−**	Suppl. Fig. 5
Ekman Pumping	**−**	**−**	**−**	Suppl. Fig. 5
MLD	+	nc	**−**	Fig. 3
SST	**−**	nc	+	Fig. 2
SSS	**−**	nc	+	Fig. 2
TA	**−**	nc	+	Fig. 2
ΔDIC_a-s_	**−**	nc	+	Suppl. Fig. 6
Chlorophyll *a*	**+**	**−**	**−**	ref. ^24^

“+” denotes an increase with positive SAM trend, “−” denotes a decrease with positive SAM trend and “nc” denotes no change. Suppl. Fig. 5 and Suppl. Fig. 6 stand for Supplementary Figure 5 and Supplementary Figure 6, respectively. Note although SSS (or TA) change was not statistically significant (*p*-values of ~0.3) at high-latitudes (60°–55° S) probably due to small sample numbers of <10 (Fig. 2b, d), its trend of decrease was consistent with that observed in entire Antarctic Zone of the Southern Ocean^60^. Thus, in this table only trends with *p*-values > 0.3 are regarded as no change

Since pH and Ω_arag_ decreased with depth (Fig. 5a, b), enhanced vertical mixing should also lead to an enhanced acidification. To determine whether changes in vertical mixing in the upper ocean can be a major contributor to a change of acidification rates, we examined changes in mixed layer depth (MLD, Fig. 3d, h, l). During a period with positive SAM trends at high-latitudes (60°–55° S), MLD showed an increasing trend (Fig. 3d), suggesting an increase in vertical mixing that will entrain more subsurface waters with low pH and Ω_arag_ into the mixed layer (Fig. 5a, b), which enhances acidification rates. In contrast, in the mid-latitude band (50°–45° S), MLD showed a decreasing trend during a positive SAM trend (Fig. 3l), suggesting a decrease in vertical mixing that will entrain less subsurface waters into the mixed layer (Fig. 5a, b), which suppresses acidification rates. However, there were almost no changes in MLD during 2001–2011 at high-latitudes and mid-latitudes or in either period in the transition zone (Fig. 3), revealing that mixing in the upper ocean has no obvious changes during these periods.

It seems that Ekman transport brings more water from the higher latitudes than from the subsurface water to the surface Antarctic Zone, since observed changes of SST and SSS at the three latitudinal zones (Fig. 2) are consistent with changes expected due to Ekman transport (Supplementary Fig. 5a; Table 1). For example, at high-latitudes (60°–55° S) during a positive SAM trend, increased equatorward Ekman transport should induce a drop in SSS (Supplementary Fig. 7), whereas increased vertical mixing should cause a rise in SSS (Supplementary Fig. 7), but in fact we observed a decrease in SSS (Fig. 2b). However, vertical mixing may still play an important role in modulating OA due to the stronger gradients of pH and Ω_arag_ in the vertical direction than in the lateral direction (Fig. 5a, b; Supplementary Table 5). For instance, vertically from Point N to Point D, salinity increased by 0.08, and pH_@7.45_ and Ω_arag@7.45_ decreased by 0.06 and 0.25 units, respectively, with pH_@7.45_ change per unit salinity of −0.72 and Ω_arag@7.45_ change per unit salinity of −3.01. Laterally, from Point S to Point N, salinity, pH_@7.45_ and Ω_arag@7.45_ increased by 0.42, 0.07 and 0.26 units, respectively, with pH_@7.45_ change per unit salinity of 0.17 and Ω_arag@7.45_ change per unit salinity of 0.62. Therefore, vertical mixing could also play an important role in modulating OA. This is further supported by a mass balance model calculation (see 'Methods' and Supplementary Table 6). Overall, given the covariation of Ekman transport and vertical mixing with SAM associated winds (Table 1) and their consistent effects on pH and Ω_arag_, they both synergistically modulated the OA rates caused by increasing atmospheric CO_2_.

Note in our paper we use vertical mixing rather broadly and mean to include convergence (i.e., downwelling) or divergence (i.e., upwelling), trend changes of which are quantified by Ekman pumping velocity (Supplementary Fig. 5c, d). We find there was a trend of decrease (increase) in Ekman pumping at the three latitudinal zones during 1991–2000 (2001–2011) (Supplementary Fig. 5c, d), but it appears that their influences on changes of pH and Ω_arag_ are minor or not observed. This can be seen, for example, from the transition zone (55°–50° S) where there were no apparent changes in wind speed, MLD or Ekman transport in either period (Fig. 3g, h; Supplementary Fig. 5a, b). During 1991–2000 when there was a tendency toward anomalous convergence (i.e., decreasing Ekman pumping, Supplementary Fig. 5c), waters with relatively low pH and Ω_arag_ from the high-latitude zone and waters with relatively high pH and Ω_arag_ from the mid-latitude zone may have been simultaneously transported to the transition zone (Fig. 5a, b), resulting in the cancellation of these effects and no net enhanced acidification during this period (Fig. 3e, f). While there was a tendency toward increasing upwelling during the period 2001–2011 (i.e., increasing Ekman pumping, Supplementary Fig. 5d), the influence of upwelling on SST, SSS, pH, and Ω_arag_ was not observed in the transition zone (Figs. 2 and 3). It indicates that the influence of upwelling on upper mixed layer was probably small and was susceptible to other processes, which needs further studies in this region.

Several processes could contribute to enhanced OA relative to atmospheric CO_2_ increase. These could include an increase in equatorward transport of high-latitude water, an increase in vertical mixing/upwelling, a decrease in biological production, and an increased gas exchange rate due to increasing wind speed. While we cannot discount the importance of changes in local air-sea CO_2_ flux and biological activity, the mass balance analysis suggests that they both only play a relatively minor role in modulating acidification induced by increasing atmospheric CO_2_ (see 'Methods'). Instead, the trend in TA (Fig. 2) and the mass balance analysis (see 'Methods') confirm that lateral and vertical transport are the dominant processes in modulating OA rates, since TA is immune to air-sea CO_2_ fluxes and is just weakly influenced by biology^28^. By these analyses and by comparing observed changes of quantities with expected influences of SAM-associated winds (Table 1), we therefore conclude that the observed enhanced acidification in the high-latitudes and suppressed acidification in the mid-latitudes are primarily attributable to wind-driven Ekman transport and vertical mixing.

## Discussion

To put our findings in a broad context, we explore the possible influence of the SAM on pH and Ω_arag_ changes in other regions of the Southern Ocean by examining CCMP (Cross-Calibrated Multi-Platform) January zonal wind trends in the Southern Ocean basin (Fig. 1b, c). We find that during positive SAM trends (1991–2000), January wind speed increased at high-latitudes (poleward of the subantarctic front, SAF), and decreased at mid-latitudes (equatorward of SAF) except in parts of the Pacific sector, similar to the patterns shown by Lovenduski and Gruber^24^. In contrast, during a period with insignificant SAM trends (2001–2011), January zonal winds generally exhibited an opposite spatial pattern—decreasing at high-latitudes and increasing at most mid-latitudes. In January, across the entire Southern Ocean wind speed trends were largely consistent with those in our study region, though there were regional differences in the location of the transitional zone between high-latitude and mid-latitude patterns (Fig. 1b, c). This suggests that our study area can represent the meridional feature of the wind change in the Southern Ocean, and that the SAM modulation of OA rates south of Tasmania is part of a SAM-regulated change spanning the whole Southern Ocean. Given the spatial distribution of pH_@7.45_ and Ω_arag@7.45_ in the entire Southern Ocean (Fig. 5), it may be inferred that during austral summer, the SAM would have substantial impacts on acidification rates in the whole Southern Ocean via wind forcing, although some heterogeneities do exist among the different Southern Ocean sectors. For instance, during 2001–2011, January zonal wind speed showed a decreasing trend in the 60°–55° S band south of Tasmania and in most other sectors in the Southern Ocean, but a weak increasing trend in the Drake Passage (68°–56° W in Fig. 1c). Accordingly, the rate of Ω_arag_ decrease during this period appeared to be somewhat greater in the Drake Passage (0.013 ± 0.003 yr^–1^ for austral summer during 2002–2015, see Munro et al.^29^) than in the high-latitude band in our study area (0.007 ± 0.005 yr^–1^). This case further supports the mechanism of wind-driven modulation of OA, although it still needs to be verified in other sectors since the pattern of MLD change south of Tasmania is not fully consistent with that in other parts of the Southern Ocean (Supplementary Fig. 5e, f).

Our study based on observational data further supports the idea that the SAM has modulating effects on the Southern Ocean CO_2_ system, mainly via wind-driven Ekman transport and vertical mixing during stratified austral summer (Supplementary Fig. 7). Studies based on ocean circulation models^30,31^ and atmospheric CO_2_ inverse methods^23^ suggest modulation of the SAM on Southern Ocean carbon uptake. This conclusion is also supported by *f*CO_2_ observations^32–34^. In contrast, using data derived by a neural network technique, Landschützer et al.^35^ find that, on an annual basis, the reinvigoration of Southern Ocean CO_2_ uptake after the early 2000s cannot be explained by the SAM-associated wind trends, because there are almost no changes in annual trends of wind between the 1990s and the 2000s (Supplementary Fig. 8). Instead, they propose a mechanism associated with a more zonally asymmetric atmospheric circulation^35^. More recently, however, using ocean circulation models, DeVries et al.^36^ find that increased Southern Ocean CO_2_ uptake in the 2000s compared to the 1990s was due to reduced upwelling (a weakened upper-ocean overturning circulation, similar to our mechanism).

There are at least two possible reasons that can explain why our results differ from those of Landschützer et al.^35^. One important reason is that there would be a large difference between seasonal trends (e.g., summer) and annual trends. To validate this, we chose the CCMP wind data^37,38^ to examine the differences between January and annual trends. We find that there are substantial differences between January and annual trends (Fig. 1 and Supplementary Fig. 8). Another reason is that the SAM and its effects have a strong seasonality, with the most pronounced influence during austral summer^19,20^. Additionally, we recognize that changes in water properties of previous winter and spring seasons may also affect summer water properties, which is not discussed in our work due to data limitations and should be concerned in future observational and modeling efforts. Therefore, our study supports SAM modulation of acidification rates during austral summer (January), but sufficient observational data are not available^27^ to elucidate the full annual influence of SAM on acidification. To resolve these issues, more observations are needed in this remote ocean for all seasons, especially during the poorly sampled austral winter.

Our work clarifies the discrepancy regarding the influence of the SAM on Southern Ocean acidification. Previous studies in this aspect are all based on models that yielded different understandings of SAM impacts. Some of these studies indicate that a positive SAM trend would not substantially affect OA or that the role of climate-driven physical changes would be minor^18,21^. While other studies argue that OA in the entire Southern Ocean would be enhanced during a positive SAM trend^22,23^ in agreement with our viewpoint, they did not reveal latitudinal differences in their studies. Therefore, our work helps improve understanding of the mechanisms of OA in the Southern Ocean, which is important for modeling atmospheric CO_2_ uptake and ecosystem responses. Overall, our work provides observational support for climatic modulation of OA, which should be taken into account in future predictions of acidification. It is most likely that climate change and variability have already been affecting the advance of OA in the global ocean via wind forcing^14^, which requires further observations.

## Methods

### Data collection and processing

Sea surface *f*CO_2_, SST, and SSS data from 17 cruises south of Tasmania (along or near Transect SR03, 60°–45° S) during 1991–2011 are used in this study (Supplementary Figs. 2–3). These data were extracted from the Surface Ocean CO_2_ Atlas (SOCAT version 2)^25^ (http://www.socat.info/) and were collected by the groups of Bronte Tilbrook, Hisayuki Y. Inoue, Nicolas Metzl, Rik Wanninkhof, and Taro Takahashi^32,39–41^. TA and DIC data from discrete seawater samples collected along Transect SR03 during December 1994–January 1995, September 1996, March 1998, October–November 2001 and April 2008 in combination with salinity, temperature, and latitude data were used to derive the TA relationship (see 'TA estimation'). Also, TA and DIC data during December 1994–January 1995 were used to calculate pH_@7.45_ and Ω_arag@7.45_ as shown in Fig. 5a, b. These data were obtained from the Global Ocean Data Analysis Project, Version 2 (GLODAPv2)^42^.

SAM index used in this study is observation-based and developed by Marshall^20^, and is available at http://www.antarctica.ac.uk/met/gjma/sam.html. CCMP wind data^37^ were chosen for examining changes in zonal wind speed because of their good data quality^38^. This product has a resolution of 0.25° and is available at http://podaac.jpl.nasa.gov/datasetlist?search=ccmp. Monthly mean MLD data with a resolution of 0.5° determined by temperature criteria from SODA v3.3.1 (Simple Ocean Data Assimilation, available at http://apdrc.soest.hawaii.edu/dods/public_data/SODA/soda_3.3.1/) were used to examine the variability of MLD during January 1991–2011.

Before calculating carbonate system parameters, we binned, averaged, and deseasonalized the surface underway data, including sea surface *f*CO_2_, SST, and SSS. For each parameter, we first binned all data points into 0.02° latitudinal bands to overcome different sampling frequencies among the cruises, calculated the average for each band, and finally took average values for the latitudinal bands of 60°–55° S, 55°–50° S and 50°–45° S, respectively as in Xue et al.^34^. For deseasonalization, we adjusted the averaged *f*CO_2_, SST, and SSS data to January values using the long-term averaged seasonal cycle obtained by Takahashi et al.^27^ (Supplementary Tables 1–3) as done by e.g., Lauvset and Gruber^43^. January was chosen because it is the month during which most data are collected (Supplementary Figs. 2–3) and also because the influence of the Antarctic ozone hole on surface climate is most pronounced during austral summer^19^. In addition, there is an evident SAM trend during this month (Fig. 1a). The averaged and deseasonalized *f*CO_2_, SST, and SSS values at 60°–55° S, 55°–50° S, and 50°–45° S bands between 142.5° and 147.5° E are shown in Fig. 2.

### TA estimation

Surface water TA within the study area was estimated using SSS (PSS), SST (°C) and latitude (Lat, in decimal degrees, negative for South latitudes) via Eq. (1):

1 $${\mathrm{TA}}\left( {{\mathrm{\mu mol}}\;{\mathrm{kg}}^{ - 1}} \right) = 35.94 \times {\mathrm{SSS}} + 0.49 \times {\mathrm{SST}} - 1.65 \times {\mathrm{Lat}} + 964.97$$

(*r*^2^ = 0.92, *n* = 346)

Equation (1) was determined via multiple linear regression using the measured data in the upper 60 dbar along Transect SR03 collected during December 1994–January 1995, September 1996, March 1998, October–November 2001 and April 2008 (see 'Data collection and processing'). A comparison between estimated TA and the measured TA yielded a root mean square error (RMSE) of ±3.5 μmol  kg^–1^ (Supplementary Fig. 4a). This is better than what was initially derived from the global equation of Lee^44^, which generates a RMSE of ±6.4 μmol kg^–1^.

To examine uncertainty associated with the TA estimation, DIC, pH, and Ω_arag_ were calculated from *f*CO_2_ derived from the observed TA and DIC and the estimated TA, using the CO2SYS program^45^ and the apparent carbonic acid dissociation constants of Mehrbach et al.^46^ as refit by Dickson and Millero^47^. The resulting RMSEs for DIC, pH, and Ω_arag_ were ±3.0 μmol kg^–1^, ±0.0010 and ±0.005, respectively, when compared with measured DIC, and calculated pH and Ω_arag_ from a measured DIC and TA pair (Supplementary Fig. 4b–d). According to the error-calculation method of Lauvset and Gruber^43^, the calculation errors of pH and Ω_arag_ are estimated to be 0.0022 and 0.010, respectively. Given the uncertainties of ±(0.005–0.01) for spectrophotometrically measured pH (refs. ^48–50^) and ±0.18 for Ω_arag_ calculated from paired measurements of carbonate parameters^51,52^, we conclude that error associated with the estimation of TA will not affect our results or conclusions.

### Calculation of pH and Ω_arag_

Surface pH on the total H^+^ concentration scale at in situ temperature and at the regional mean temperature of 7.45 °C was calculated using the CO2SYS program^45^, with inputs of measured surface *f*CO_2_ and estimated TA (Fig. 2) and climatological phosphate and silicate concentrations (Supplementary Table 4, though nutrient effects on pH and Ω_arag_ are small). The apparent carbonic acid dissociation constants of Mehrbach et al.^46^ as refit by Dickson and Millero^47^ were used, as recommended by Chen et al.^53^ for polar ocean waters. For calculating Ω_arag_ (=[CO_3_^2−^] × [Ca^2+^] / Ksp_aragonite_), carbonate ion concentration ([CO_3_^2−^]) was also calculated using the CO2SYS program^45^. The calcium ion concentration ([Ca^2+^]) was calculated from salinity (0.01026 / 35 × salinity [mol kg^–1^]) based on the conservative behavior of [Ca^2+^] to salinity^54^, and the apparent solubility product of aragonite (Ksp_aragonite_) was calculated after Mucci^52^. Also, pH_@7.45_ and Ω_arag@7.45_ in the upper 400 dbar along Transect SR03 during December 1994–January 1995 (Fig. 5a, b) and climatological values of surface pH_@7.45_ and Ω_arag@7.45_ in January (Fig. 5c, d)^9^ were calculated from the TA and DIC data, respectively.

### Quantification of ocean acidification rates

First, we calculated a weighted three-year running mean (1:2:1) for the SAM time series and found that the January SAM trend showed a clear shift in 2000 (gray line in Fig. 1a), splitting the data into two decades (i.e., 1991–2000 vs. 2001–2011). Then, following the definition of OA^5^, rates of OA are characterized by rates of pH and Ω_arag_ change with time (i.e., slopes in Fig. 3), which were obtained using ordinary least squares linear regressions over each of the two decades of interest. To obtain the rates of decrease of pH and Ω_arag_ due solely to atmospheric CO_2_ increase, we used the CO2SYS program^45^ to calculate pH and Ω_arag_ from constant TA and increasing *f*CO_2_, i.e., we held TA, SSS, and SST constant at their 1991 values while allowing surface water *f*CO_2_ to increase at the same rate as atmospheric CO_2_ observed at the GCO (Cape Grim, Tasmania) atmospheric CO_2_ measurement station (ftp://aftp.cmdl.noaa.gov/data/trace_gases/co2/flask/; Fig. 2c). Similarly, changes in DIC and the difference between TA and DIC ([TA−DIC]) due solely to the increase in atmospheric CO_2_ were calculated. Given that air-sea CO_2_ exchange affects DIC but not TA, changes in [TA−DIC] that are due solely to changes in atmospheric CO_2_ will have the same amplitude as the changes in DIC but will be of opposite sign (i.e., different direction of change). Rates of pH and Ω_arag_ change without atmospheric CO_2_ increase (i.e., excluding the effects of increasing atmospheric CO_2_, Fig. 4) were the observed rates of pH_@__in_ _situ_ and Ω_arag@__in_ _situ_ change subtracting their rates predicted from atmospheric CO_2_ increase alone (shown by gray dashed lines in Fig. 3).

### Thermal influences on OA rates

The thermal influences on changes of pH and Ω_arag_ due to temperature changes are relatively minor. This is because there were no substantial changes in SST during the study period, although during a positive SAM trend (1991–2000) SST showed a decrease trend at 60°–55° S band, and an increase trend at 50°–45° S band (Fig. 2a, f, k). During 1991–2000 for the 60°–55° S band, although there was a decrease trend in SST of 0.08 °C yr^–1^ (Fig. 2a), thus tending to increase pH (ref. ^55^), the observed rate of pH_@__in_ _situ_ decrease was still faster than the rate attributable solely to atmospheric CO_2_ increase (gray dashed line, Fig. 3a). Comparing the rates of decrease for pH_@__in_ _situ_ (Fig. 3a) and pH_@7.45_ (Supplementary Fig. 6a) during this period shows that the effect of decreasing SST only partly counteracted the pH decreases. During 1991–2000 for the 50°–45° S band, the difference between the pH_@__in_ _situ_ and pH_@7.45_ trends is not statistically significant (Fig. 3i and Supplementary Fig. 6k). Compared to pH, Ω_arag_ is relatively insensitive to temperature changes, with surface Ω_arag@__in_ _situ_ and Ω_arag@7.45_ showing almost the same variability and rates of change throughout (Fig. 3 and Supplementary Fig. 6).

### Mass balance analysis of Ekman transport and vertical mixing

For convenience and effectiveness of discussion, we introduce a combined property, the difference between the concentrations of TA and DIC, i.e., [TA−DIC]. Unlike pH and Ω_arag_, [TA−DIC] is a conservative quantity composed of two conservative parameters, and hence is suited for analysis of water mass mixing. The [TA−DIC] approximates closely the concentration of carbonate ions ([CO_3_^2–^]) by definition^56^ and can be used as a proxy for pH and Ω_arag@in situ_^57^. In our dataset, [TA−DIC] correlates well with pH_@7.45_ and with Ω_arag@__in_ _situ_, with a correlation coefficient of *r* of nearly one (Supplementary Fig. 9). Thus, [TA−DIC] can be used to assess the changing effect of a process on OA rate.

To examine the relative contribution of Ekman transport vs. vertical mixing on OA rates, we use salinity and TA as conservative tracers to resolve changes in TA and DIC due to changes in these two processes. In the following, we take the case at high-latitudes during the 1991–2000 positive SAM trend as an example, and estimate the relative contribution of these two processes. We selected three points along transect SR03 to be compared including surface point S (South) and point N (North) and deep point D under N (Fig. 5 and Supplementary Table 5). To derive quantitatively the amounts of TA, DIC, and [TA−DIC] changes caused by changes in lateral transport and vertical mixing during a positive SAM period, we consider the changes of salinity (*S*) and TA in surface waters at point N:

2 $$\Delta {S}_{\mathrm{E}} + \Delta {S}_{\mathrm{V}} = \Delta {S}$$

3 $$\Delta {\mathrm{TA}}_{\mathrm{E}} + \Delta {\mathrm{TA}}_{\mathrm{V}} = \Delta {\mathrm{TA}}$$

where the sign “Δ” denotes changes of a parameter; and subscripts “E” and “V” denote Ekman transport and vertical mixing, respectively. Note that the mass balances are built upon the changes of salinity and TA during the period but not on the absolute amount due to lateral and vertical transports. Also, we neglect the influence of change in precipitation–evaporation balance as its influence on TA and DIC is small and similar, and thus its influence on [TA−DIC] is negligibly small.

Based on gradients per salinity change between Points S and N (lateral), and Points D and N (vertical) (Supplementary Table 5), we

4 $$\Delta {\mathrm{TA}}_{\mathrm{E}} = 50.95 \times \Delta {S}_{\mathrm{E}}$$

5 $$\Delta {\mathrm{TA}}_{\mathrm{V}} = 84.34 \times \Delta {S}_{\mathrm{V}}$$

Thus, Eq. (3) can be rewritten as

6 $$50.95 \times \Delta {S}_{\mathrm{E}} + 84.34 \times {\Delta S}_{\mathrm{V}} = {\mathrm{\Delta TA}}$$

Since Δ*S* and ΔTA are known during the positive SAM period (slope values in Fig. 2b, d), through Eq. (2) and (6), we obtain Δ*S*_E_ = −0.023 yr^−1^ and Δ*S*_V_ = 0.006 yr^−1^ during the positive SAM period. Thus, based on the gradients of TA and DIC shown in Supplementary Table 5, the respective contribution of Ekman transport and vertical mixing on TA, DIC, and [TA−DIC] can be calculated (Supplementary Table 6

$$\Delta {\mathrm{TA}}_{\mathrm{E}} = -0.023 \times 50.95 = - 1.17\;{\mathrm{\mu mol}}\;{\mathrm{kg}}^{ - {\mathrm{1}}}\;{\mathrm{yr}}^{ - {\mathrm{1}}},$$

$$\Delta {\mathrm{DIC}}_{\mathrm{E}} = -0.023 \times \left( { - 18.96} \right) = 0.44\;{\mathrm{\mu mol}}\;{\mathrm{kg}}^{ - 1}\;{\mathrm{yr}}^{ - 1},$$

$$\Delta \left[ {{\mathrm{TA{-DIC}}}} \right]_{\mathrm{E}} = - 1.61\;{\mathrm{\mu mol}}\; \mathrm{kg}^{ - 1}\;{\mathrm{yr}}^{ - 1};$$

$$\Delta {\mathrm{TA}}_{\mathrm{V}} = 0.006 \times 84.34 = 0.51\;{\mathrm{\mu mol}}\;{\mathrm{kg}}^{ - {\mathrm{1}}}\;{\mathrm{yr}}^{ - {\mathrm{1}}},$$

$$\Delta {\mathrm{DIC}}_{\mathrm{V}} = 0.006 \times 366.27 = 2.20\;{\mathrm{\mu mol}}\;{\mathrm{kg}}^{ - {\mathrm{1}}}\;{\mathrm{yr}}^{ - {\mathrm{1}}},$$

$$\Delta [{\mathrm{TA{-DIC}}} ]_{\mathrm{V}} = - 1.69\;{\mathrm{\mu mol}}\;{\mathrm{kg}}^{ - {\mathrm{1}}}\;{\mathrm{yr}}^{ - {\mathrm{1}}}$$

Our calculations show that at high-latitudes during the 1991–2000 positive SAM trend the contribution of Ekman transport and vertical mixing on OA rates (as Δ[TA–DIC]) are likely on the same order of magnitudes (Supplementary Table 6).

### Impacts of air-sea CO_2_ flux and biological activity on OA

Similar to salinity and TA, we have the mass balance of DIC

7 $$\Delta {\mathrm{DIC}}_{\mathrm{E}} + \Delta {\mathrm{DIC}}_{\mathrm{V}} + \Delta {\mathrm{DIC}}_{\mathrm{A}} + \Delta {\mathrm{DIC}}_{\mathrm{B}} = \Delta {\mathrm{DIC}}$$

Here “A” and “B” denote changes in air-sea exchange and biological activity, respectively.

Based on this mass balance and the observed ΔDIC and calculated ΔDIC_E_ and ΔDIC_V_, the total contribution to DIC change and thus [TA−DIC] change from air-sea gas exchange and biology is obtained (Δ[TA−DIC]_A_ + Δ[TA−DIC]_B_ = 1.30 µmol kg^−1^ yr^−1^), which is less than the effect by physical transports associated with changes in wind speed (Δ[TA−DIC]_E_ + Δ[TA−DIC]_V_ = −3.3 µmol kg^−1^ yr^−1^) (Supplementary Table 6). This result is consistent with that obtained by analyzing the trend changes of each process (Table 1). For example, during 1991–2000 at high-latitudes both decreasing air-sea CO_2_ flux and increasing biological production^24^ should result in a decrease in DIC (Supplementary Fig. 6; Table 1) and thus an increase in [TA−DIC], which can partly cancel out OA. In contrast, during this period increasing Ekman transport and vertical mixing (Table 1) should enhance OA. Comparing with observed enhancement of OA rates (Fig. 3a, b) indicates that Ekman transport and vertical mixing play a dominant role in modulating OA rates.

Further, Δ[TA−DIC]_A_ and Δ[TA−DIC]_B_ during 1991–2000 at high-latitudes can be calculated (Supplementary Table 6). When the time of 30 (or 100) days is considered for CO_2_ uptake each summer, ΔDIC_A_ and Δ[TA−DIC]_A_ would be −0.29 (or −0.97) μmol kg^−1^ yr^−1^ and 0.29 (or 0.97) μmol kg^−1^ yr^−1^, respectively (slope values in Supplementary Fig. 6e), and thus ΔDIC_B_ and Δ[TA−DIC]_B_ would be −1.01 (or −0.33) μmol kg^−1^ yr^−1^ and 1.01 (or 0.33) μmol kg^−1^ yr^−1^, respectively (Supplementary Table 6). Despite the fact that we cannot fully constrain the relative contribution between air-sea CO_2_ flux and biology, during the 1991–2000 positive SAM period at high-latitudes, biological carbon uptake induced an increase in [TA−DIC], reducing OA, which is consistent with the increasing biological production reported previously^24^. Note that in this paper we discuss the decadal changes of parameters or processes rather than seasonal changes of them. For example during 1991–2000 at high-latitudes the decrease in air-sea CO_2_ flux (Supplementary Fig. 6d) and the increase in biological production^24^ (characterized by chlorophyll) both should decrease DIC and thus increase [TA−DIC], reducing OA, although on seasonal timescale, for example, during January air-sea CO_2_ flux (absorbing CO_2_) will increase DIC and reduce pH and Ω_arag_, and biological carbon uptake will decrease DIC and increase pH and Ω_arag_.

### Data availability

Sea surface *fCO*_2_ data can be obtained from the Surface Ocean CO_2_ Atlas (SOCAT version 2) (http://www.socat.info/) and the data that support the findings of this study are available from the corresponding author upon reasonable request.

## Electronic supplementary material

Supplementary Information

Peer Review File

## References

[1] Fabry VJ, McClintock JB, Mathis JT, Grebmeier JM (2009). Ocean acidification at high latitudes: the bellweather. Oceanography.

[2] Orr JC (2005). Anthropogenic ocean acidification over the twenty-first century and its impact on calcifying organisms. Nature.

[3] McNeil BI, Matear RJ (2008). Southern Ocean acidification: a tipping point at 450-ppm atmospheric CO_2_. Proc. Natl Acad. Sci..

[4] Midorikawa T (2012). Decreasing pH trend estimated from 35-year time series of carbonate parameters in the Pacific sector of the Southern Ocean in summer. Deep Sea Res. Part I.

[5] Cooley, S. R., Mathis, J. T., Yates, K. K. & Turley, C. Frequently asked questions about ocean acidification. U.S. Ocean Carbon and Biogeochemistry Program and the UK Ocean Acidification Research Programme. Version 2. 24 September 2012. www.whoi.edu/OCB-OA/FAQs (2012).

[6] Doney SC, Fabry VJ, Feely RA, Kleypas JA (2009). Ocean acidification: the other CO_2_ problem. Annu. Rev. Mar. Sci..

[7] Bednaršek N (2012). Extensive dissolution of live pteropods in the Southern Ocean. Nat. Geosci..

[8] Moy AD, Howard WR, Bray SG, Trull TW (2009). Reduced calcification in modern Southern Ocean planktonic foraminifera. Nat. Geosci..

[9] Takahashi T, Sutherland SC, Chipman DW, Goddard JG, Ho C (2014). Climatological distributions of pH, *p*CO_2_, total CO_2_, alkalinity, and CaCO_3_ saturation in the global surface ocean, and temporal changes at selected locations. Mar. Chem..

[10] Cai, W.-J. et al. Acidification of subsurface coastal waters enhanced by eutrophication. *Nat. Geosci.* **4**, 766–770 (2011).

[11] Dore JE, Lukas R, Sadler DW, Church MJ, Karl DM (2009). Physical and biogeochemical modulation of ocean acidification in the central North Pacific. Proc. Natl Acad. Sci..

[12] Feely RA, Sabine CL, Hernandez-Ayon JM, Ianson D, Hales B (2008). Evidence for upwelling of corrosive “acidified” water onto the continental shelf. Science.

[13] Gruber N (2012). Rapid progression of ocean acidification in the California current system. Science.

[14] Turi, G., Lachkar, Z., Gruber, N. & Münnich, M. Climatic modulation of recent trends in ocean acidification in the California Current System. *Environ. Res. Lett.* **11**, 014007 (2016).

[15] Yamamoto-Kawai M, McLaughlin FA, Carmack EC, Nishino S, Shimada K (2009). Aragonite undersaturation in the Arctic Ocean: effects of ocean acidification and sea ice melt. Science.

[16] Brewer PG (2009). A changing ocean seen with clarity. Proc. Natl Acad. Sci..

[17] Qi D (2017). Increase in acidifying water in the western Arctic Ocean. Nat. Clim. Change.

[18] Hauri, C., Friedrich, T. & Timmermann, A. Abrupt onset and prolongation of aragonite undersaturation events in the Southern Ocean. *Nat. Clim. Change* **6**, 172–176 (2016).

[19] Thompson DWJ (2011). Signatures of the Antarctic ozone hole in Southern Hemisphere surface climate change. Nat. Geosci..

[20] Marshall GJ (2003). Trends in the southern annular mode from observations and reanalyses. J. Clim..

[21] Conrad CJ, Lovenduski NS (2015). Climate driven variability in the Southern Ocean carbonate system. J. Clim..

[22] Lenton A (2009). Stratospheric ozone depletion reduces ocean carbon uptake and enhances ocean acidification. Geophys. Res. Lett..

[23] Le Quéré C (2007). Saturation of the Southern Ocean CO_2_ sink due to recent climate change. Science.

[24] Lovenduski NS, Gruber N (2005). Impact of the Southern Annular Mode on Southern Ocean circulation and biology. Geophys. Res. Lett..

[25] Bakker D (2014). An update to the Surface Ocean CO_2_ Atlas (SOCAT version 2). Earth Syst. Sci. Data.

[26] Sokolov S, Rintoul SR (2002). Structure of Southern Ocean fronts at 140 E. J. Mar. Syst..

[27] Takahashi T (2009). Climatological mean and decadal change in surface ocean *p*CO_2_, and net sea-air CO_2_ flux over the global oceans. Deep Sea Res. Part II.

[28] Xue, L. et al. Sea surface carbon dioxide at the Georgia time series site (2006-2007): air-sea flux and controlling processes. *Prog. Oceanogr.* **140**, 14–26 (2016).

[29] Munro, D. R. et al. Recent evidence for a strengthening CO_2_ sink in the Southern Ocean from carbonate system measurements in the Drake Passage (2002–2015). *Geophys. Res. Lett.* **42**, 7623–7630 (2015).

[30] Lenton A, Matear RJ (2007). Role of the Southern Annular Mode (SAM) in southern ocean CO_2_ uptake. Glob. Biogeochem. Cycles.

[31] Lovenduski NS, Gruber N, Doney SC, Lima ID (2007). Enhanced CO_2_ outgassing in the Southern Ocean from a positive phase of the Southern Annular Mode. Glob. Biogeochem. Cycles.

[32] Metzl N (2009). Decadal increase of oceanic carbon dioxide in Southern Indian Ocean surface waters (1991–2007). Deep Sea Res. Part II.

[33] Fay AR, McKinley GA (2013). Global trends in surface ocean *p*CO_2_ from in situ data. Glob. Biogeochem. Cycles.

[34] Xue L, Gao L, Cai WJ, Yu W, Wei M (2015). Response of sea surface fugacity of CO_2_ to the SAM shift south of Tasmania: regional differences. Geophys. Res. Lett..

[35] Landschützer P (2015). The reinvigoration of the Southern Ocean carbon sink. Science.

[36] DeVries T, Holzer M, Primeau F (2017). Recent increase in oceanic carbon uptake driven by weaker upper-ocean overturning. Nature.

[37] Atlas R (2011). A cross-calibrated, multiplatform ocean surface wind velocity product for meteorological and oceanographic applications. Bull. Am. Meteorol. Soc..

[38] Wanninkhof R (2014). Relationship between wind speed and gas exchange over the ocean revisited. Limnol. Oceanogr.: Methods.

[39] Brévière E, Metzl N, Poisson A, Tilbrook B (2006). Changes of the oceanic CO_2_ sink in the Eastern Indian sector of the Southern Ocean. Tellus B.

[40] Borges AV, Tilbrook B, Metzl N, Lenton A, Delill B (2008). Inter-annual variability of the carbon dioxide oceanic sink south of Tasmania. Biogeosciences.

[41] Yoshikawa-Inoue H, Ishii M (2005). Variations and trends of CO_2_ in the surface seawater in the Southern Ocean south of Australia between 1969 and 2002. Tellus B.

[42] Olsen A (2016). The Global Ocean Data Analysis Project version 2 (GLODAPv2) – an internally consistent data product for the world ocean. Earth Syst. Sci. Data.

[43] Lauvset SK, Gruber N (2014). Long-term trends in surface ocean pH in the North Atlantic. Mar. Chem..

[44] Lee K (2006). Global relationships of total alkalinity with salinity and temperature in surface waters of the world’s oceans. Geophys. Res. Lett..

[45] Lewis, E. & Wallace, D. W. R. *Program Developed for CO*_2_ *System Calculations.* Report No. ORNL/CDIAC 105 (Carbon Dioxide Information Analysis Center, Oak Ridge National Laboratory, Oak Ridge, 1998).

[46] Mehrbach C, Culberson CH, Hawley JE, Pytkowicz RM (1973). Measurement of the apparent dissociation constants of carbonic acid in seawater at atmospheric pressure. Limnol. Oceanogr..

[47] Dickson AG, Millero FJ (1987). A comparison of the equilibrium constants for the dissociation of carbonic acid in seawater media. Deep Sea Res. Part A. Oceanogr. Res. Pap..

[48] Friis K, Kortzinger A, Wallace DWR (2004). Spectrophotometric pH measurement in the ocean: requirements, design, and testing of an autonomous charge-coupled device detector system. Limnol. Oceanogr.: Methods.

[49] DeGrandpre MD (2014). Considerations for the measurement of spectrophotometric pH for ocean acidification and other studies. Limnol. Oceanogr.: Methods.

[50] Bockmon EE, Dickson AG (2015). An inter-laboratory comparison assessing the quality of seawater carbon dioxide measurements. Mar. Chem..

[51] Sutton AJ (2016). Using present-day observations to detect when anthropogenic change forces surface ocean carbonate chemistry outside preindustrial bounds. Biogeosciences.

[52] Mucci A (1983). The solubility of calcite and aragonite in seawater at various salinities, temperatures, and one atmosphere total pressure. Am. J. Sci..

[53] Chen B, Cai WJ, Chen L (2015). The marine carbonate system of the Arctic Ocean: assessment of internal consistency and sampling considerations, summer 2010.. Marine Chem..

[54] Riley JP, Tongudai M (1967). The major cation/chlorinity ratios in sea water. Chem. Geol..

[55] Gieskes JM (1969). Effect of Temperature on the pH of Seawater. Limnol. Oceanogr..

[56] Broecker, W. S. & Peng, T. H. *Tracers in the Sea* (The Lamont-Doherty Geological Observatory, Palisades, 1982).

[57] Xue, L., Cai, W.-J., Sutton, A. J. & Sabine, C. Sea surface aragonite saturation state variations and control mechanisms at the Gray’s Reef time-series site off Georgia, USA (2006–2007). *Marine Chem.* **195**, 27–40 (2017).

[58] Orsi AH, Whitworth III T, Nowlin Jr WD (1995). On the meridional extent and fronts of the Antarctic Circumpolar Current. Deep Sea Res. Part I: Oceanogr. Res. Pap..

[59] Schlitzer, R. Ocean Data View v.4.7.10. http://odv.awi.de (2017).

[60] Palter JB, Galbraith ED (2014). Cessation of deep convection in the open Southern Ocean under anthropogenic climate change. Nat. Clim. Change.

