# Supplementary material for: Climatic modulation of surface acidification rates through summertime wind forcing in the Southern Ocean
Source: Nat Commun. 2018 Aug 13;9:3240. doi: 10.1038/s41467-018-05443-7 (PMC6089918; doi:10.1038/s41467-018-05443-7)
Supplement: Supplementary file 1 — Supplementary Information [file 41467_2018_5443_MOESM1_ESM.pdf]

Climatic modulation of surface acidification rates through summertime wind forcing in the Southern Ocean

Liang Xue[1, 2], Wei-Jun Cai[3, *], Taro Takahashi[4], Libao Gao [1, 2], Rik Wanninkhof[5], Meng Wei [1, 2], Kuiping Li[1, 2], Lin Feng[1, 2], and Weidong Yu[1, 2]

[1]First Institute of Oceanography, State Oceanic Administration, Qingdao 266061, China

[2]Laboratory for Regional Oceanography and Numerical Modeling, Qingdao National Laboratory for Marine Science and Technology, Qingdao 266237, China

[3]School of Marine Science and Policy, University of Delaware, Newark, DE 19716, USA

[4]Lamont-Doherty Earth Observatory of Columbia University, Palisades, NY 10964, USA

[5]NOAA Atlantic Oceanographic and Meteorological Laboratory, Miami, FL 33149, USA

*To whom correspondence should be addressed: email: wcai@udel.edu; Tel: 1-302-831-2839

This file includes:

Supplementary Figures 1-9

Supplementary Tables 1-6

Supplementary References

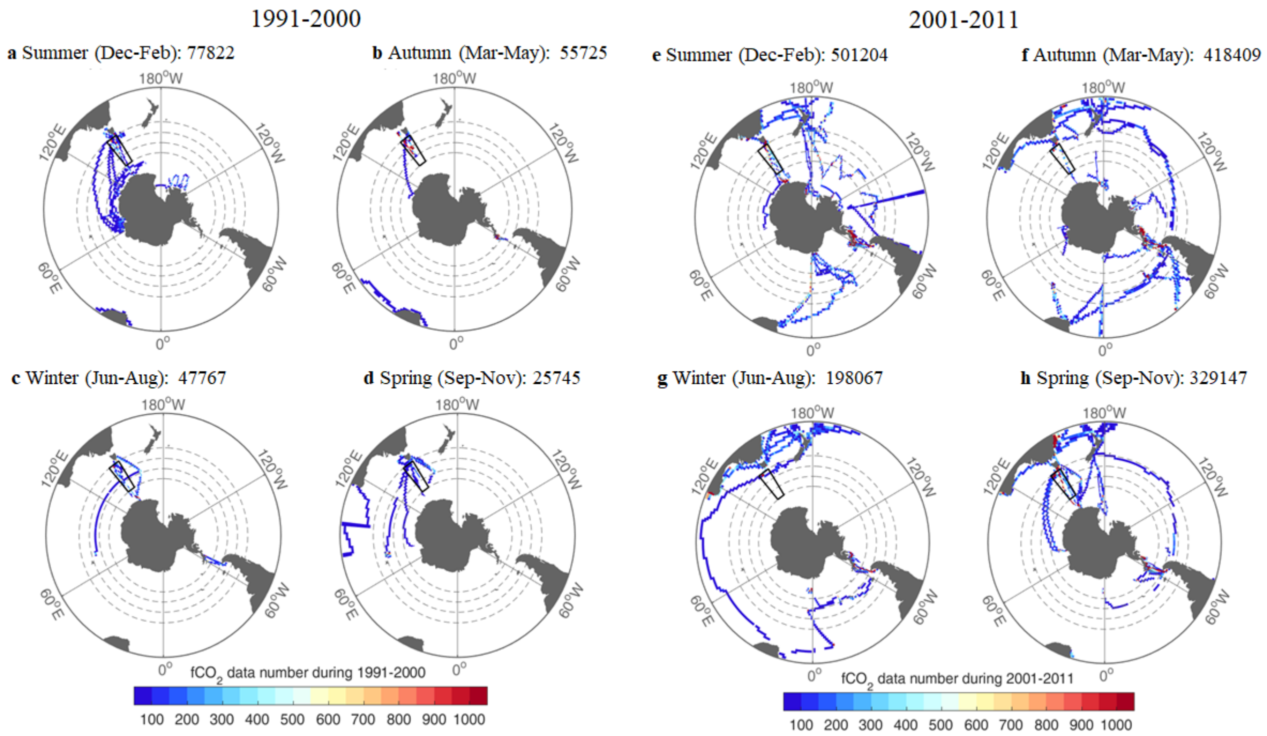

Supplementary Figure 1. Number of observed sea surface *f*CO_2 data in the Southern Ocean. a-d, during austral summer (**a**), autumn (**b**), winter (**c**) and spring (**d**) during 1991–2000, and **e-f,** during austral summer (**e**), autumn (**f**), winter (**g**) and spring (**h**) during 2001-2011. The color bars show the number of observed sea surface *f*CO_2 data in each grid (1° × 1°). These map were based on the Surface Ocean CO_2 Atlas (SOCAT) (http://www.socat.info/)[1]. Only data with “good” flag are selected.

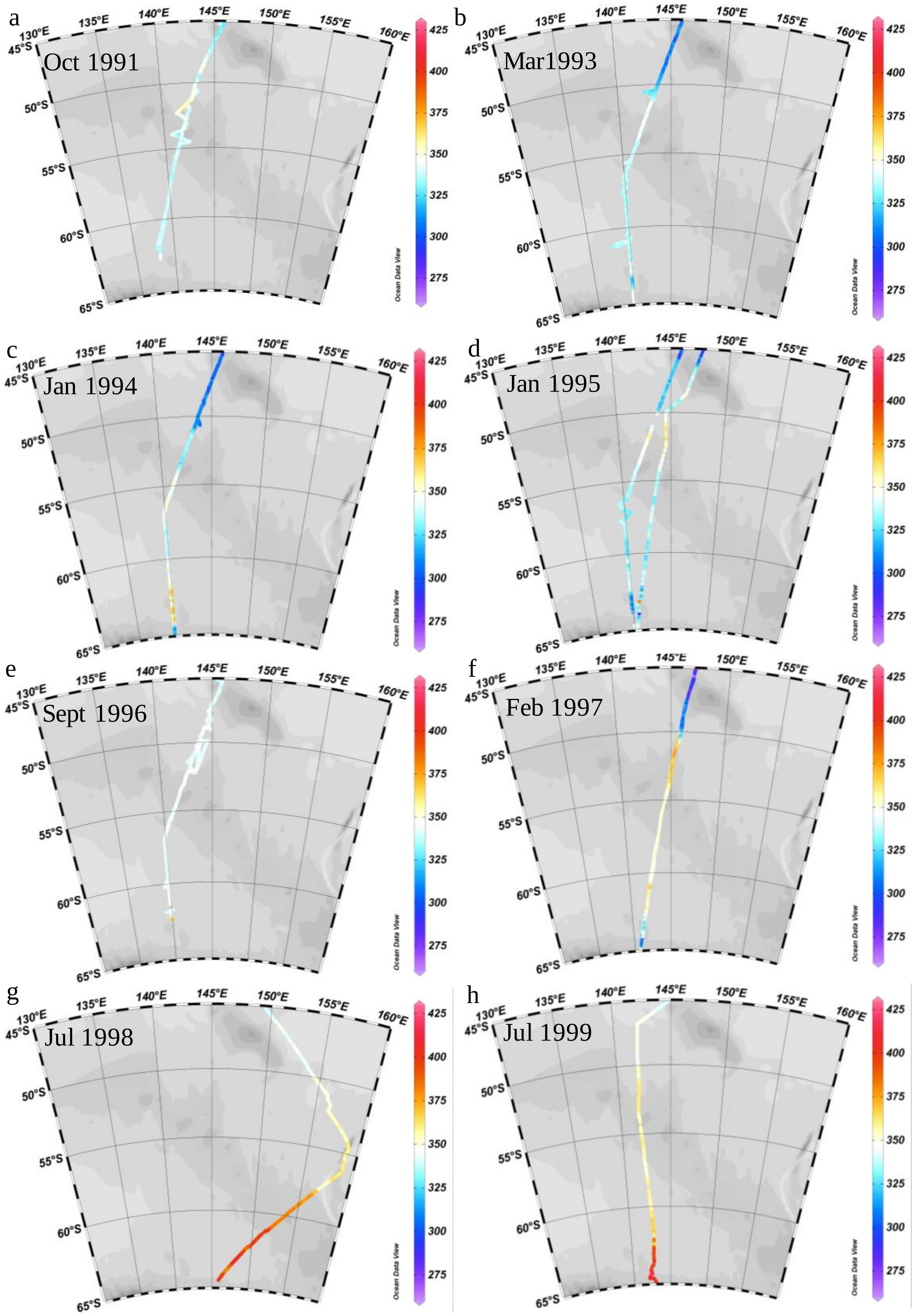
a
Oct 1991
b
Mar1993
c
Jan 1994
d
Jan 1995
e
Sept 1996
f
Feb 1997
g
Jul 1998
h
Jul 1999
130°E
135°E
140°E
145°E
150°E
155°E
160°E
45°S
50°S
55°S
60°S
65°S
425
400
375
350
325
300
275
Ocean Data View

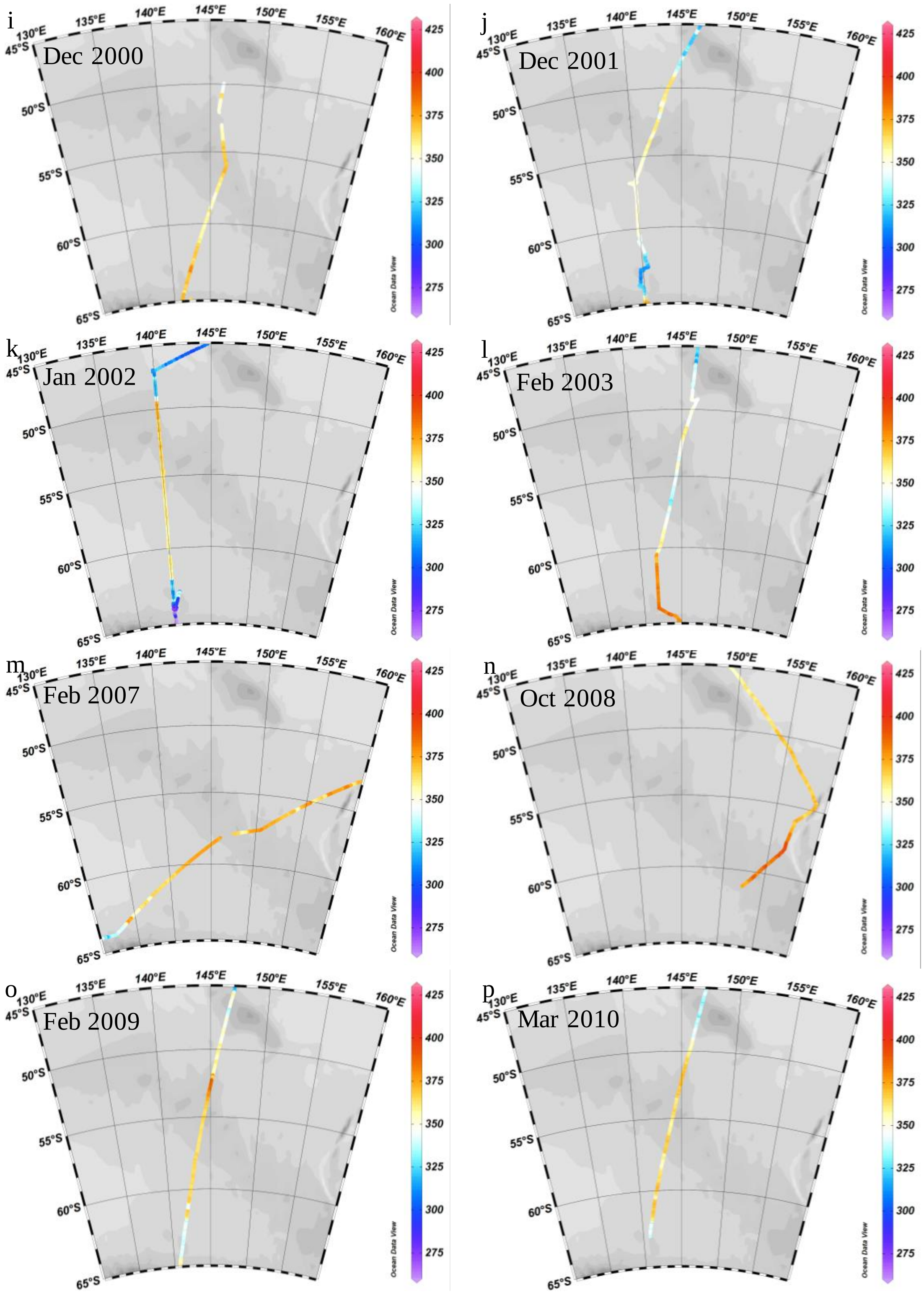
i
Dec 2000
j
Dec 2001
k
Jan 2002
l
Feb 2003
m
Feb 2007
n
Oct 2008
o
Feb 2009
p
Mar 2010
130°E
135°E
140°E
145°E
150°E
155°E
160°E
45°S
50°S
55°S
60°S
65°S
425
400
375
350
325
300
275
Ocean Data View

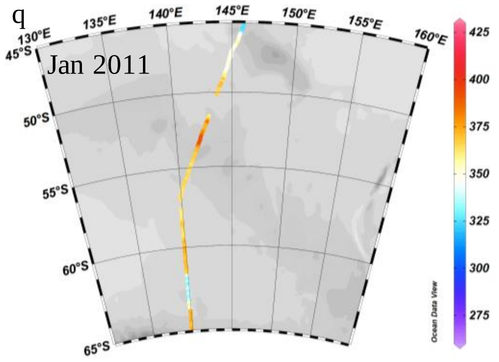

Supplementary Figure 2. Cruise tracks and associated sea surface *f*CO_2 values during the 1991–2011 expeditions south of Tasmania (a–q). The color bars denote sea surface *f*CO_2 (units of μatm). These data were extracted from the Surface Ocean CO_2 Atlas (SOCAT) (http://www.socat.info/)[1]. Supplementary Figure 2 is plotted using Ocean Data View (odv_4.7.10_w64 version)[2].

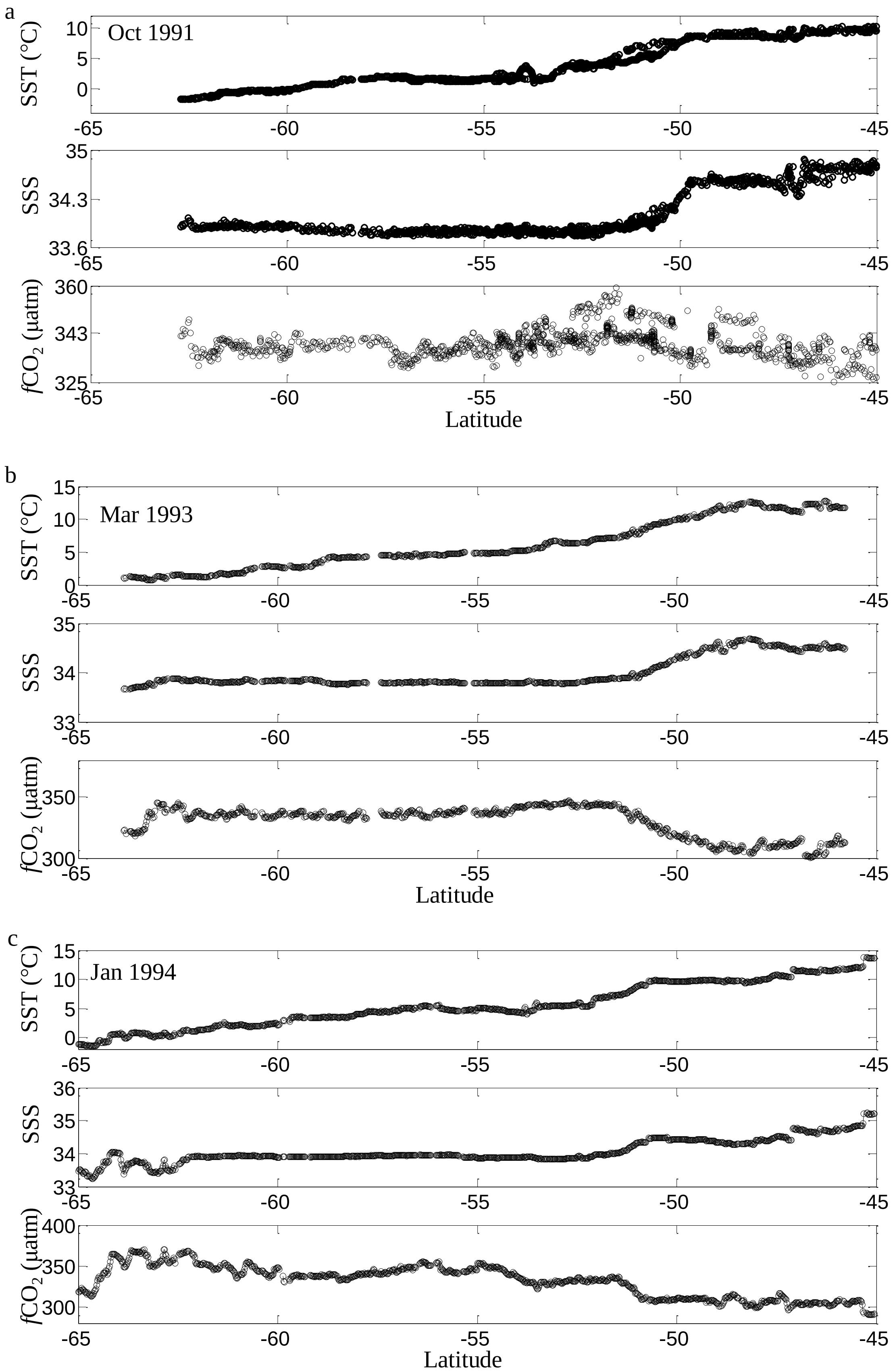
a
Oct 1991
SST (°C)
SSS
fCO2 (µatm)
Latitude
b
Mar 1993
SST (°C)
SSS
fCO2 (µatm)
Latitude
c
Jan 1994
SST (°C)
SSS
fCO2 (µatm)
Latitude

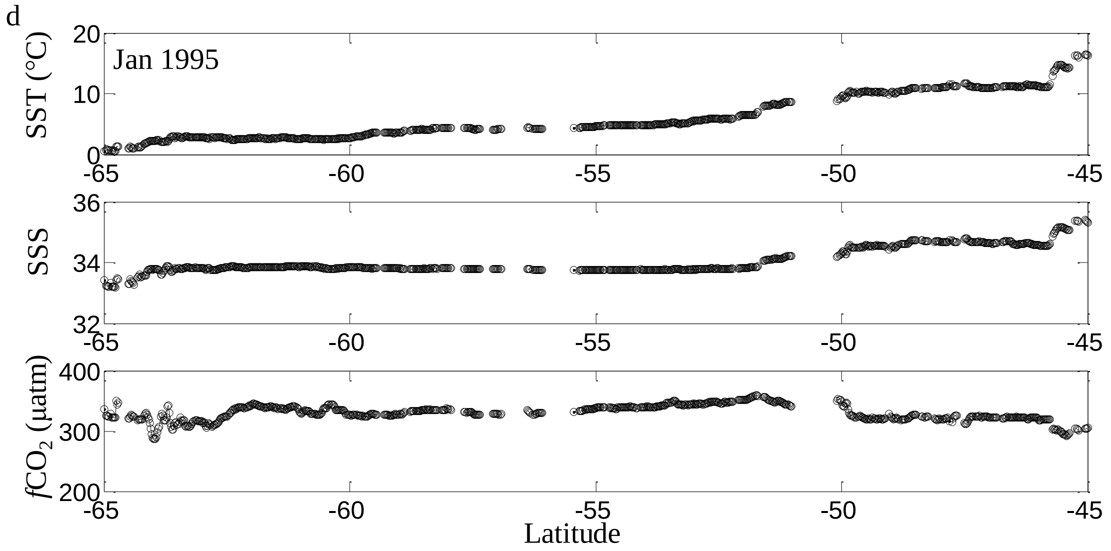
d
Jan 1995
SST (°C)
SSS
fCO_2 (µatm)
Latitude

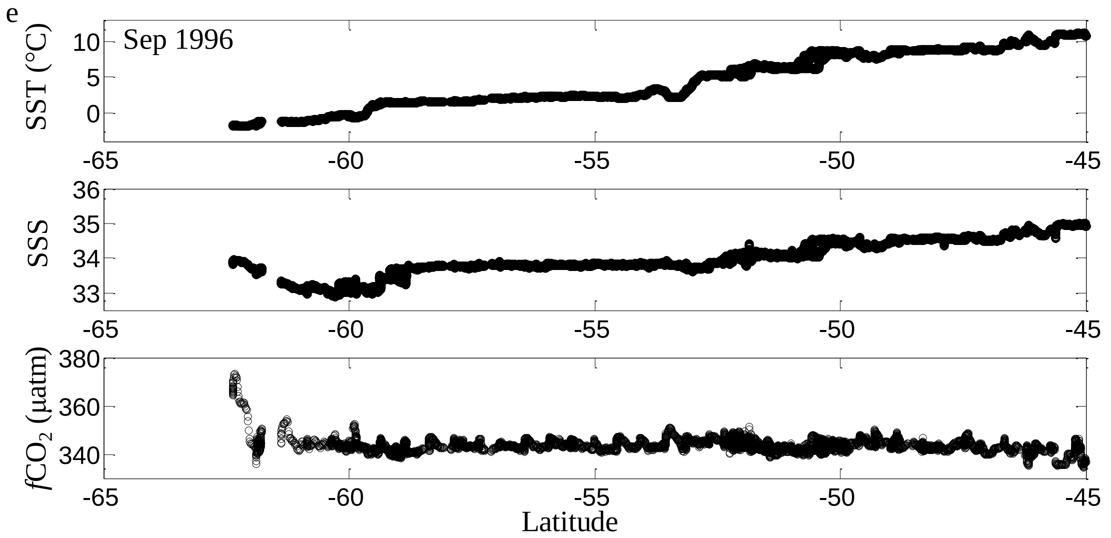
e
Sep 1996
SST (°C)
SSS
fCO_2 (µatm)
Latitude

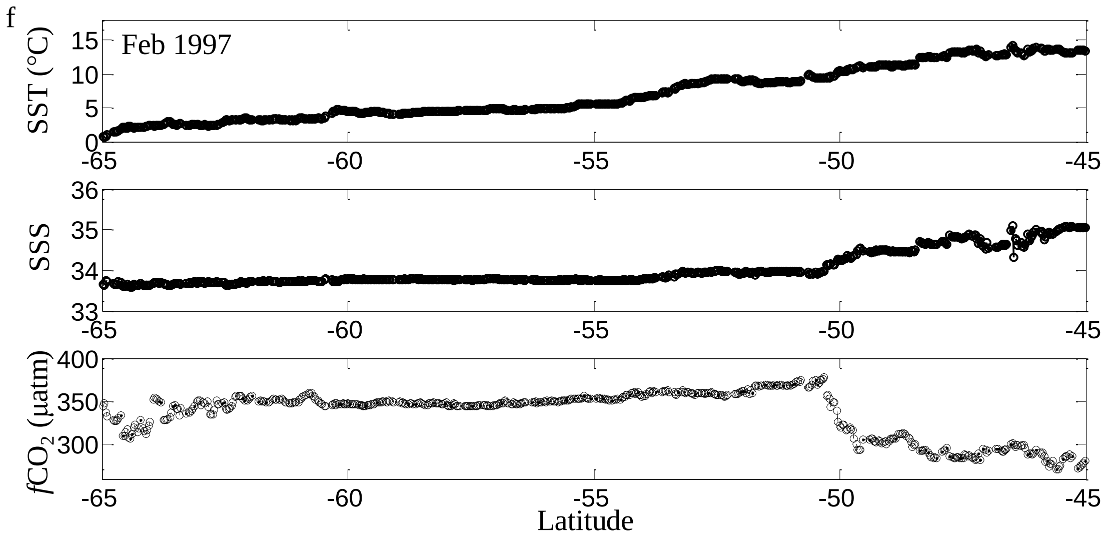
f
Feb 1997
SST (°C)
SSS
fCO_2 (µatm)
Latitude

g

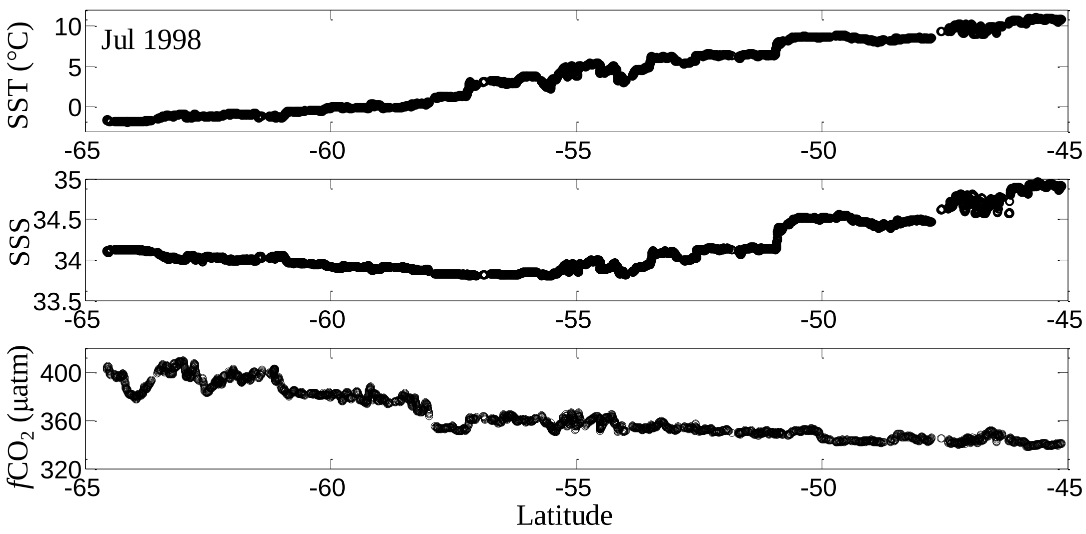
Jul 1998
SST (°C)
SSS
fCO2 (µatm)
Latitude

h

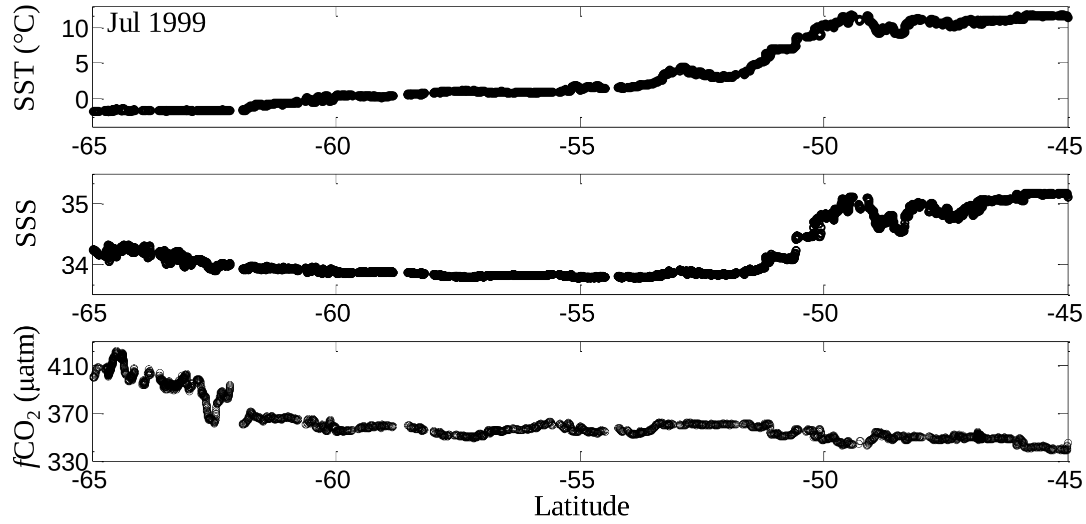
Jul 1999
SST (°C)
SSS
fCO2 (µatm)
Latitude

i

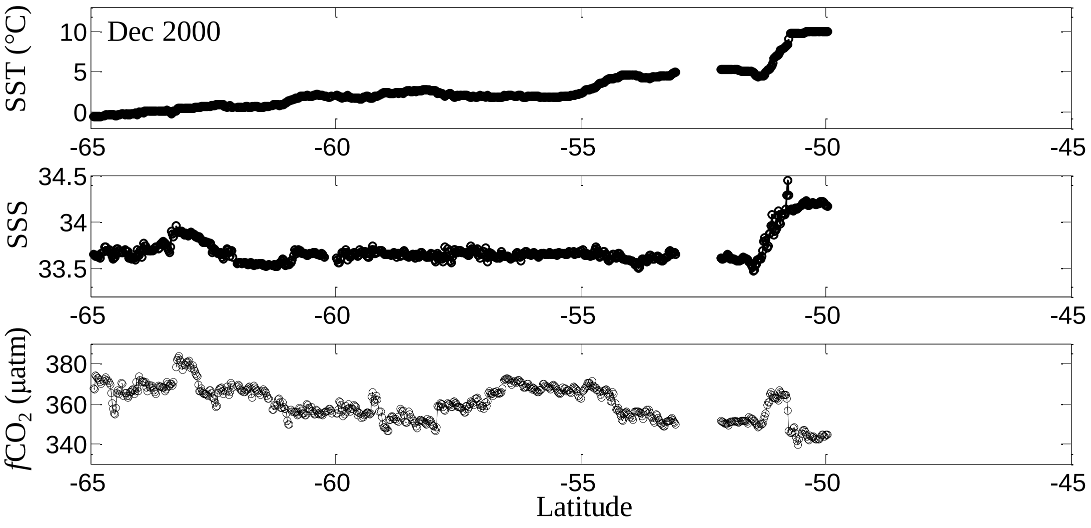
Dec 2000
SST (°C)
SSS
fCO2 (µatm)
Latitude

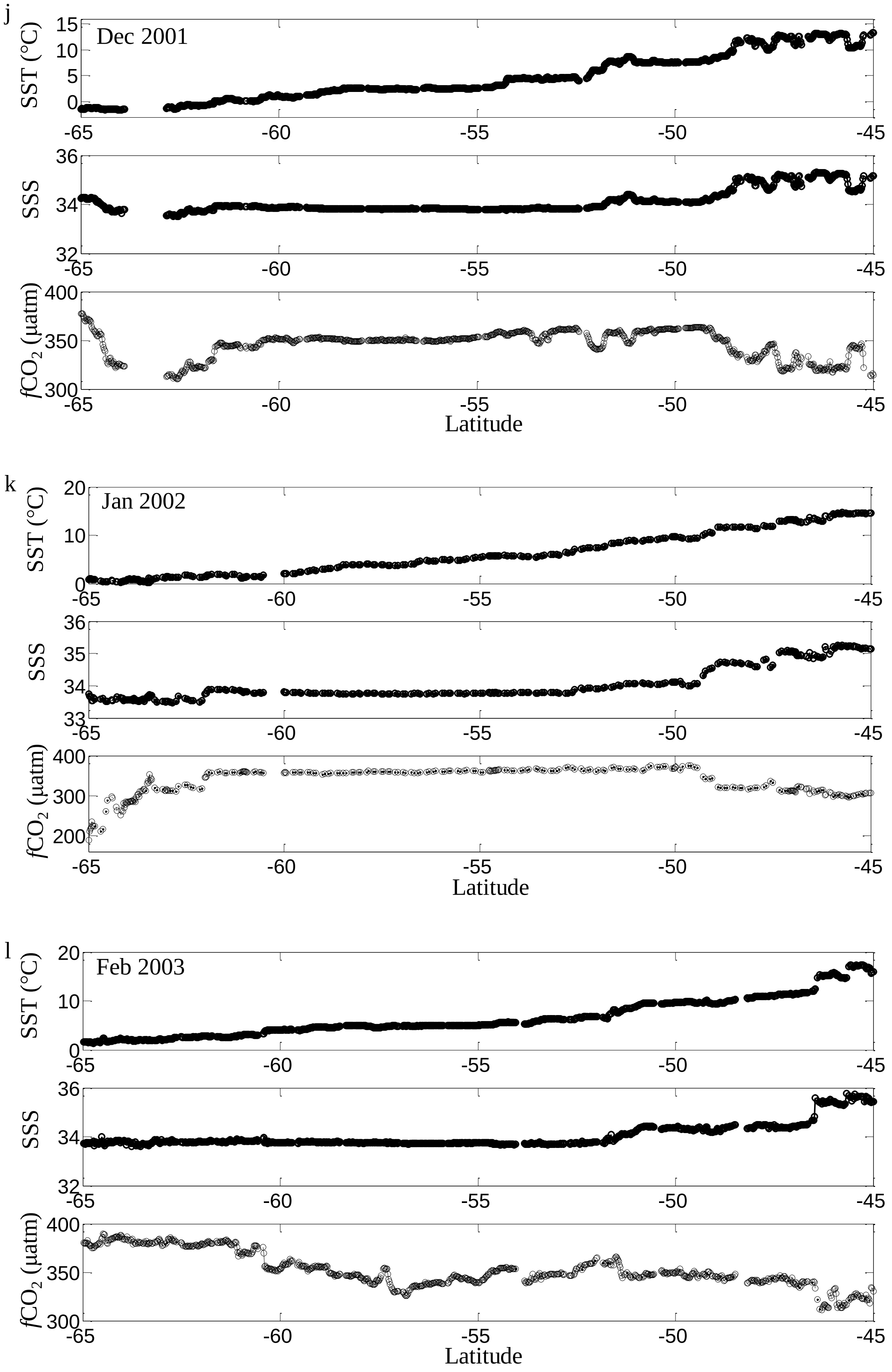
j
Dec 2001
SST (°C)
SSS
fCO2 (µatm)
Latitude
k
Jan 2002
SST (°C)
SSS
fCO2 (µatm)
Latitude
l
Feb 2003
SST (°C)
SSS
fCO2 (µatm)
Latitude

m

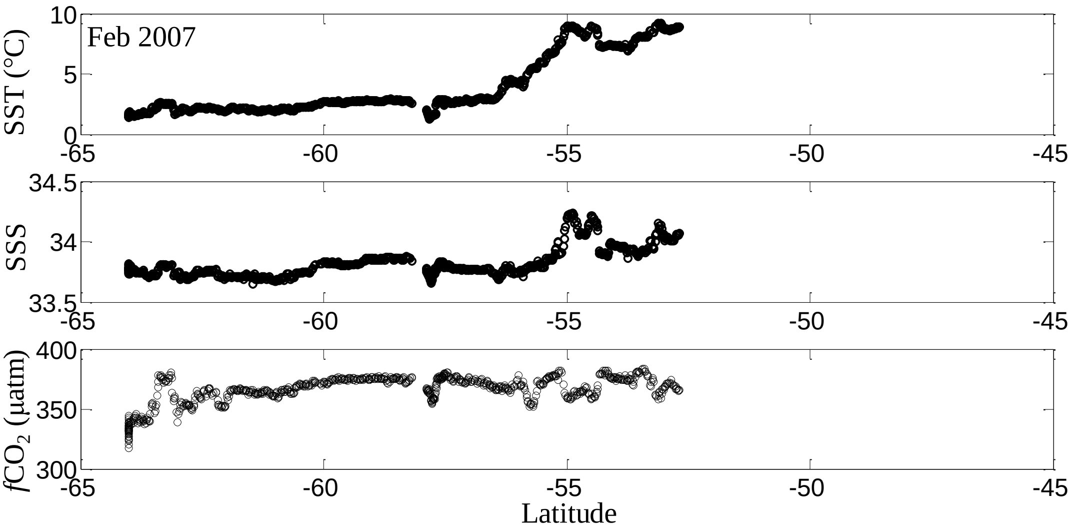
Feb 2007
SST (°C)
10
5
0
-65
-60
-55
-50
-45
SSS
34.5
34
33.5
fCO2 (µatm)
400
350
300
Latitude

n

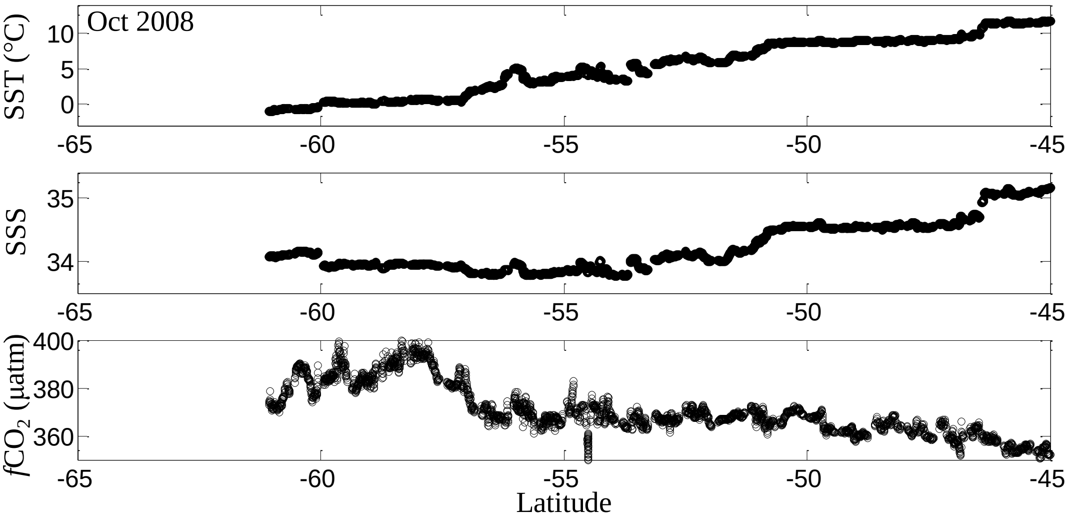
Oct 2008
SST (°C)
10
5
0
-65
-60
-55
-50
-45
SSS
35
34
fCO2 (µatm)
400
380
360
Latitude

o

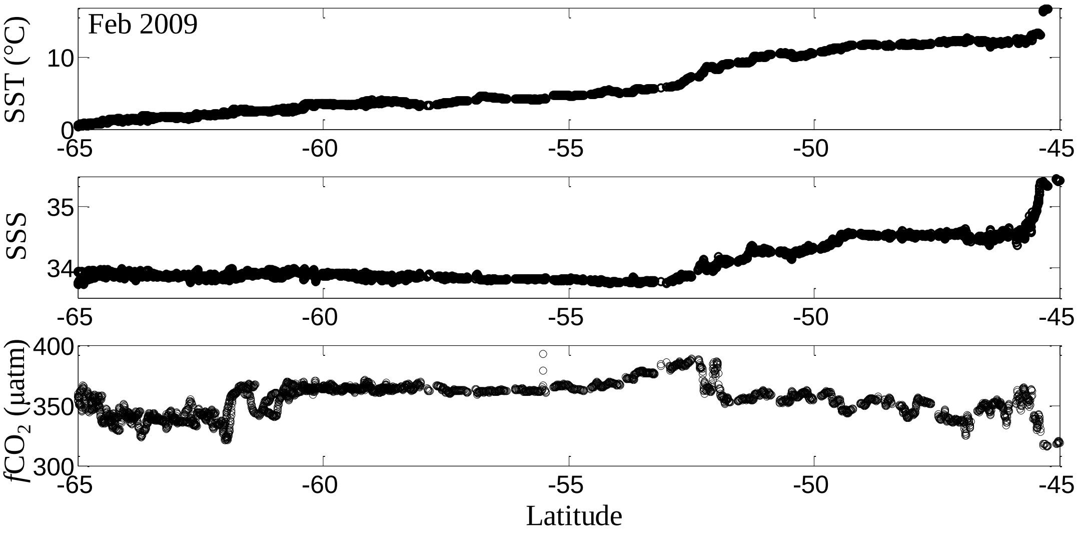
Feb 2009
SST (°C)
10
0
-65
-60
-55
-50
-45
SSS
35
34
fCO2 (µatm)
400
350
300
Latitude

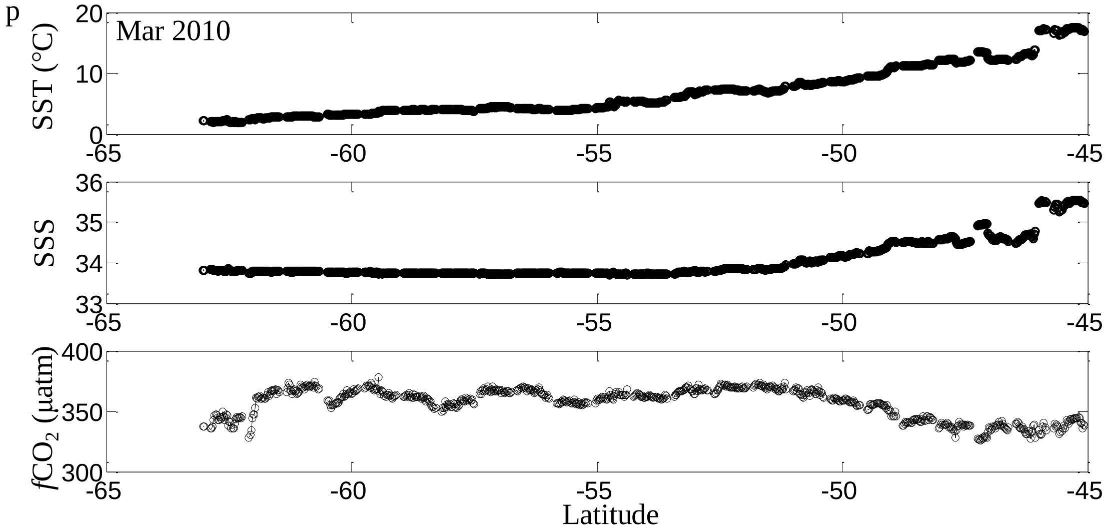

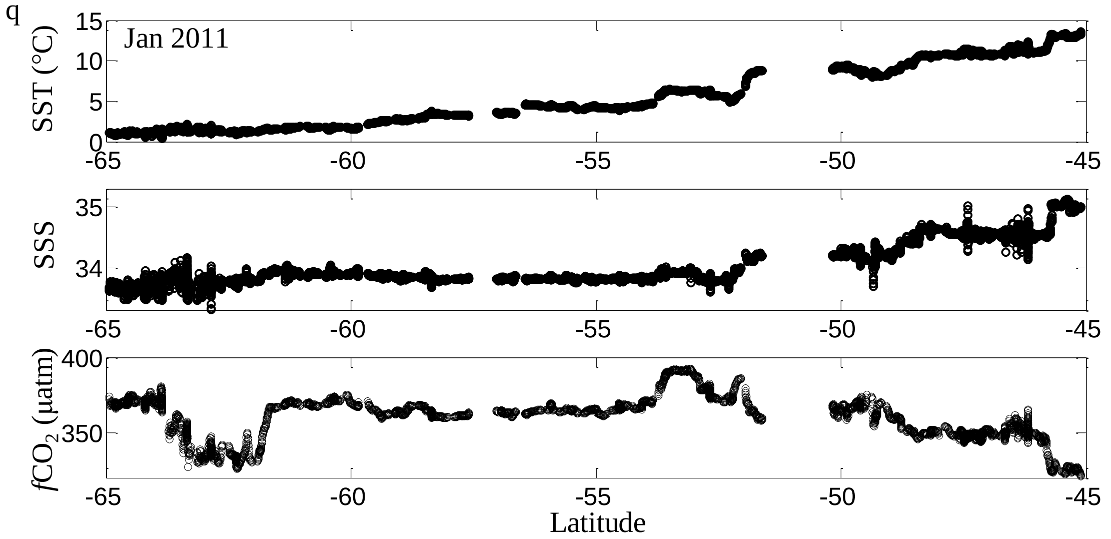

Supplementary Figure 3. Meridional distribution of SST, SSS, and surface *f*CO_2 south of Tasmania from 1991 to 2011 (a-q). Their tracks can be found in Supplementary Figure 2.

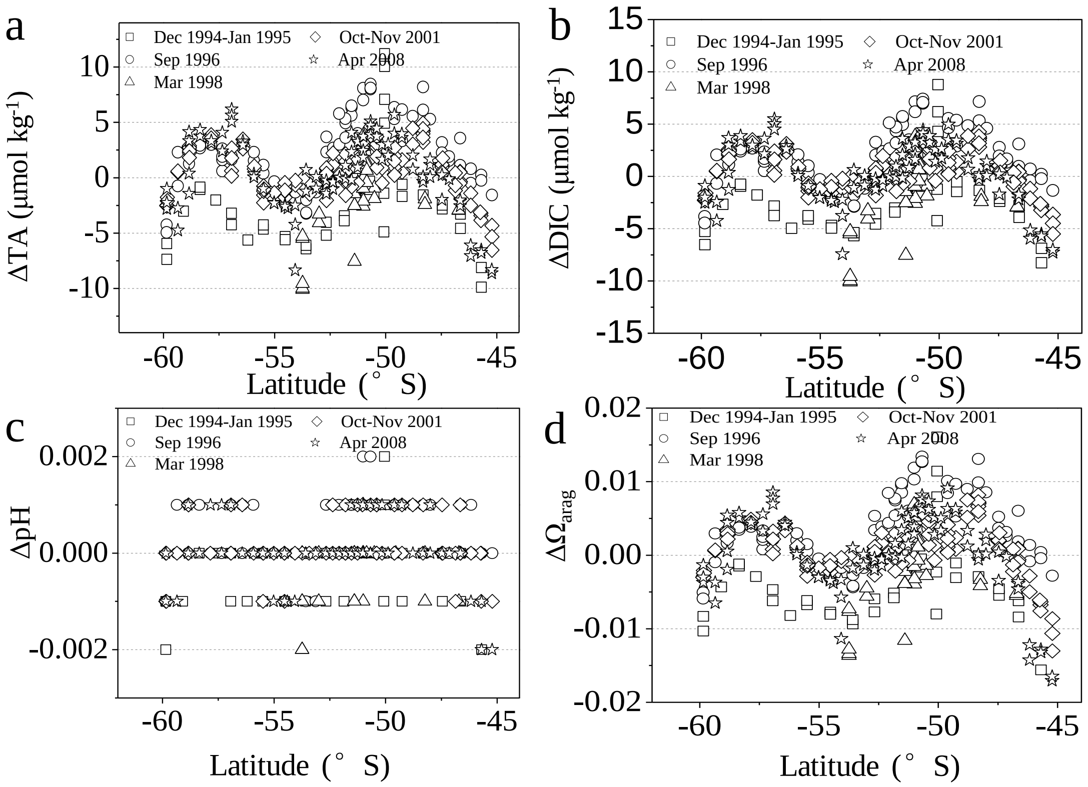

Supplementary Figure 4. Comparison between measured and estimated carbonate parameters along Transect SR03. **a**, Difference between measured and estimated TA ($\Delta TA = TA_{estimated} - TA_{measured}$). **b**, Difference between measured and estimated DIC ($\Delta DIC = DIC_{estimated} - DIC_{measured}$). **c**, Difference ($\Delta$pH) between pH calculated from paired measurements of DIC and TA and pH calculated from measured DIC and estimated TA. **d**, Difference ($\Delta\Omega_{arag}$) between Ω_{arag} calculated from paired measurements of DIC and TA and Ω_{arag} calculated from measured DIC and estimated TA. Refer to Methods for related calculations.

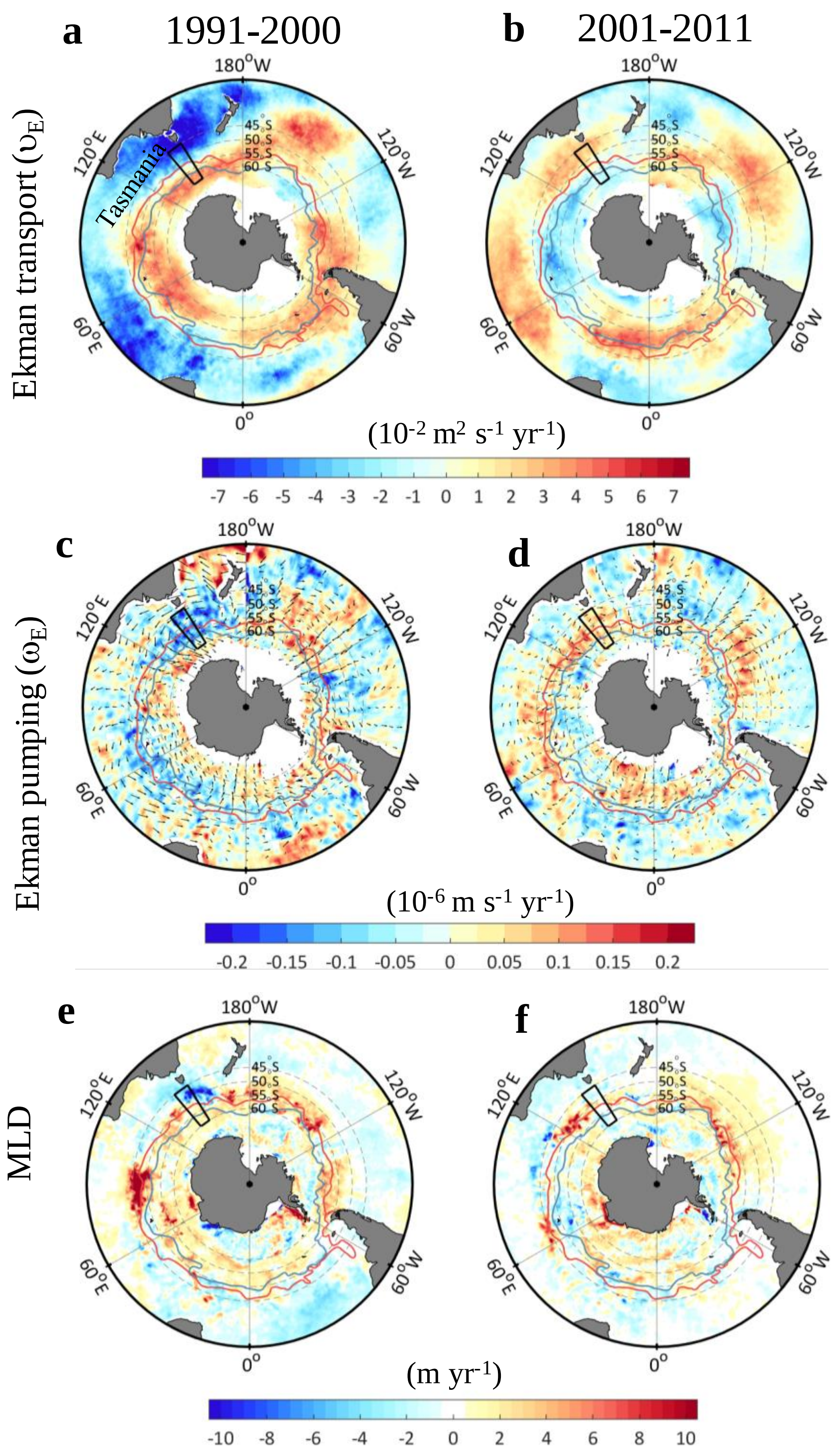

Supplementary Figure 5. January trends in meridional Ekman transport (υ_E), Ekman pumping velocity (ω_E) and mixed layer depth (MLD). **a-b**, trends in meridional Ekman transport during two periods of 1991-2000 (**a**) and 2001-2011 (**b**), **c-d**, trends in Ekman pumping during two periods of 1991-2000 (**c**) and 2001-2011 (**d**), and **e-f**, trends in MLD during two periods of 1991-2000 (**e**) and 2001-2011 (**f**). In (**c**) and (**d**), trends of 10-m wind (vectors) are shown. The northward Ekman transport ($\upsilon_E = -\frac{1}{\rho f}\tau_x$) and the Ekman pumping velocity ($\omega_E = \frac{1}{\rho f}\left[\frac{\partial \tau_x}{\partial y} - \frac{\partial \tau_y}{\partial x}\right]$) were obtained from the surface wind stress components τ_x (zonal) and τ_y (meridional). Here ρ is the reference density of 1030 kg m^{-3} and f is the Coriolis parameter. In (**c**) and (**d**), the blue color denotes a convergence trend (downwelling) and the red color a divergence trend (upwelling).

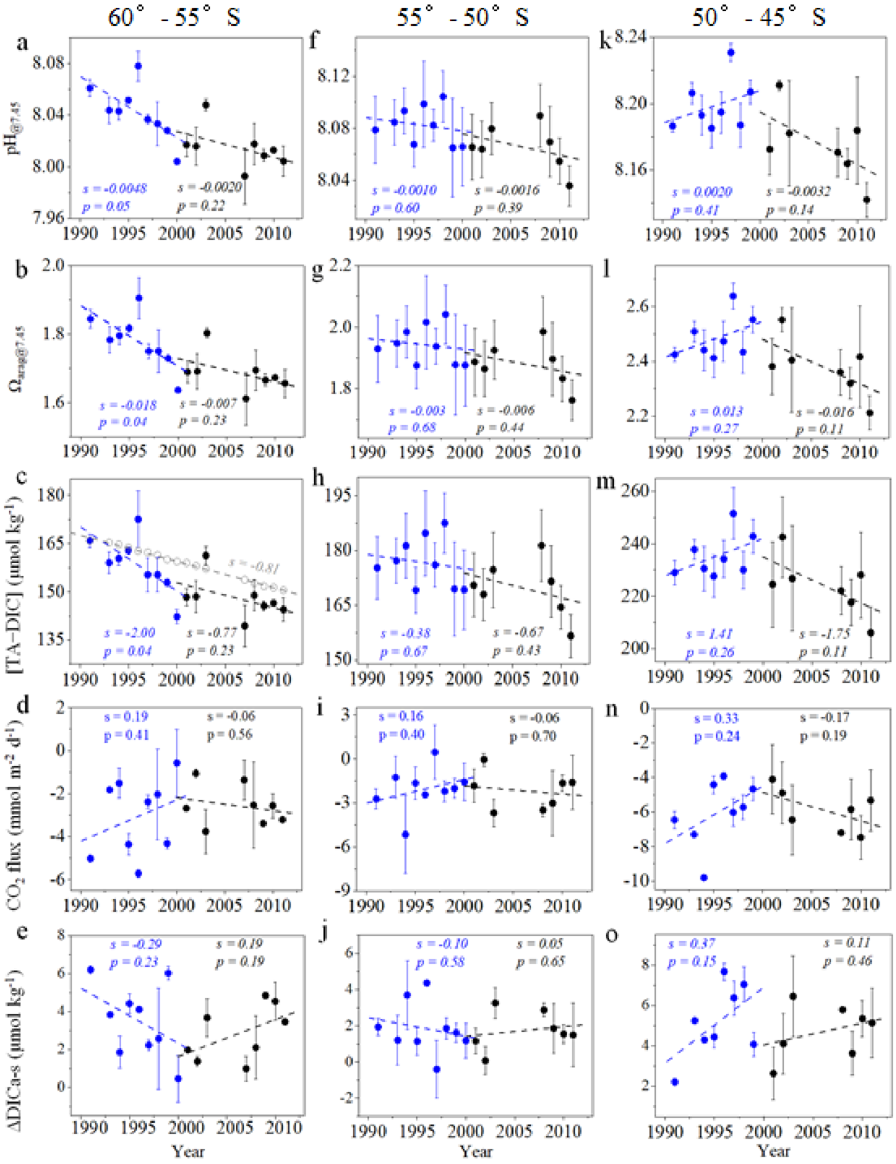

Supplementary Figure 6. Temporal variability in pH$_{@7.45}$, Ω$_{arag@7.45}$, [TA−DIC], air-sea CO_2 flux and ΔDIC$_{a\text{-}s}$ in three latitudinal bands. a-e, sea surface pH at the regional mean temperature of 7.45 ℃ (**a**, pH$_{@7.45}$), Ω$_{arag}$ at the regional mean temperature of 7.45 ℃ (**b**, Ω$_{arag@7.45}$), difference between TA and DIC (**c**, [TA−DIC]), air-sea CO_2 flux (d), and DIC gains due to atmospheric CO_2 uptake (e, ΔDIC$_{a\text{-}s}$) at 60°–55° S. Panels **(f–j)** show the same parameters but at 55°–50° S; panels **(k–o)** also show the same parameters but at 50°–45° S. The vertical bars show one standard deviation, which reflects the spatial variability within each latitudinal band. Linear regression analyses were performed for the periods 1991–2000 (blue) and 2001–2011 (black). Slopes (*s*) and *p* values of the regression analyses are also shown. A trend of *p*-value < 0.1 is regarded as statistically significant (90% confidence interval) due to the small sample numbers (<10). Air-sea CO_2 flux was calculated based on the parameterization of gas transfer velocity by Wanninkhof[3, 4]. ΔDIC$_a$ (ΔDIC$_{a\text{-}s}$ = F*t/(MLD*D)) was calculated from air-sea CO_2 fluxes (F), mixed layer depth (MLD), seawater density (D) and time (t). MLD can be seen in Fig. 3 in the main text. During calculation of ΔDIC$_{a\text{-}s}$, t = 30 days is taken.

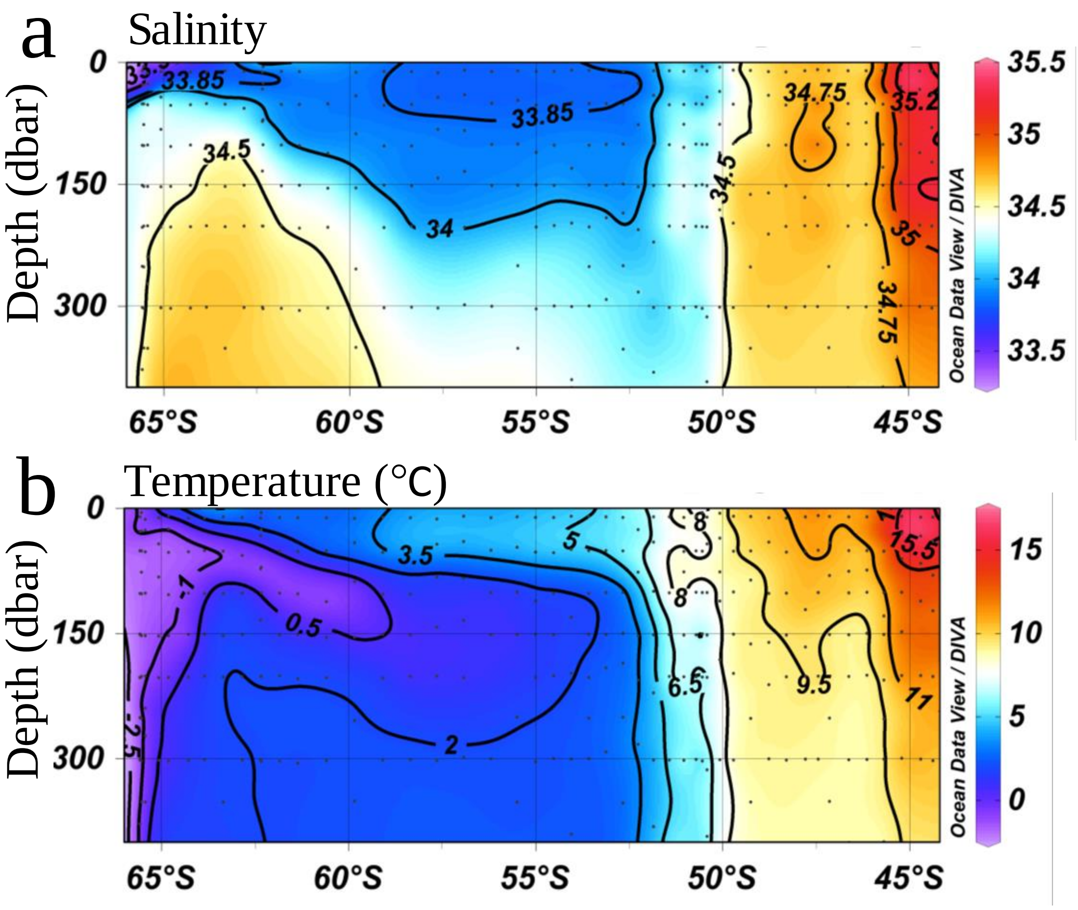

Supplementary Figure 7. Vertical distribution of salinity (a) and temperature (b) in the upper 400 dbar along Transect SR03 south of Tasmania during January 1995. Supplementary Figure 7 is plotted using Ocean Data View (odv_4.7.10_w64 version)[2].

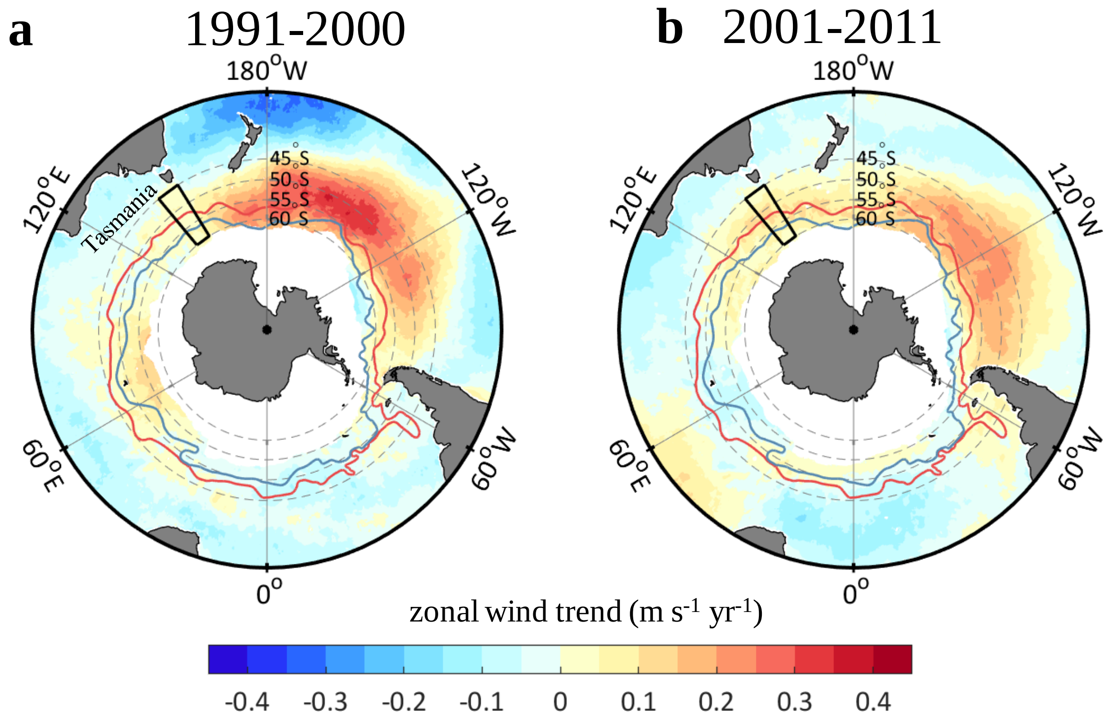

Supplementary Figure 8. Annual change rates of zonal wind speed in the Southern Ocean. **a**, during 1991-2000 and **b**, during 2001-2011. Change rates of zonal wind speed were calculated using an ordinary least squares linear regression in each grid (0.25°×0.25°) based on the Cross-Calibrated Multi-Platform (CCMP) product[5] (http://podaac.jpl.nasa.gov/datasetlist?search = ccmp). The red and blue curves show the mean positions of the subantarctic front (SAF) and the polar font (PF)[6], respectively. The black rectangle outlines the study area south of Tasmania.

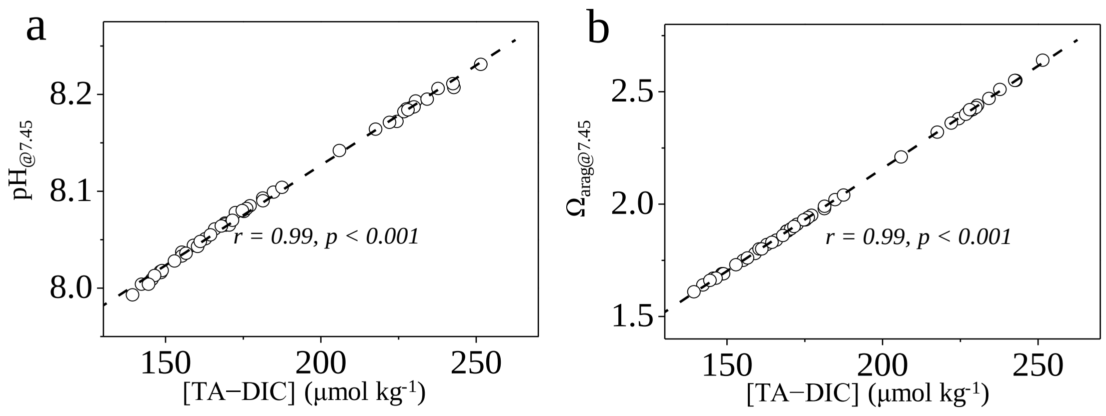

Supplementary Figure 9. Correlations between acidification parameters and [TA−DIC]. a-b, $pH_{@7.45}$ **(a)** and $\Omega_{arag@7.45}$ **(b)**. The data points are the mean values during each cruise of 1991-2011 within each latitudinal bands of 60°−55° S, 55°−50° S and 50°−45° S as show in Fig. 3 in the main text.

Supplementary Table 1. Climatological surface fCO_2, SST and SSS from January to December in the region (60°–56° S, 142.5°–147.5° E) calculated based on the climatological data by Takahashi et al.[7].

month	fCO_2 (μatm)	SST (°C)	SSS
Jan	352±7	3.42±0.92	33.73±0.05
Feb	356±3	3.93±0.87	33.82±0.01
Mar	352±4	3.54±0.89	33.86±0.005
Apr	352±2	2.71±0.95	33.84±0.02
May	356±2	1.98±0.95	33.90±0.06
Jun	358±4	1.38±0.93	33.84±0.07
Jul	369±10	0.94±0.90	33.85±0.04
Aug	376±18	0.61±0.81	33.84±0.03
Sep	380±22	0.49±0.79	33.78±0.06
Oct	366±20	0.64±0.82	33.92±0.07
Nov	361±5	1.14±0.89	33.94±0.07
Dec	352±6	2.22±0.98	33.89±0.06

Supplementary Table 2. Climatological surface fCO_2, SST and SSS from January to December in the region (52° S, 142.5°–147.5° E) calculated based on the climatological data by Takahashi et al.[7]

month	fCO_2 (μatm)	SST (°C)	SSS
Jan	350±0	7.73±0.77	34.20±0.01
Feb	350±3	7.90±0.73	34.12±0.01
Mar	344±0	7.61±0.75	34.12±0.02
Apr	357±2	6.95±0.74	34.02±0.02
May	341±1	6.48±0.72	34.16±0.14
Jun	349±2	5.98±0.78	33.91±0.02
Jul	355±0	5.66±0.86	34.11±0.06
Aug	359±1	5.39±0.91	34.16±0.02
Sep	358±1	5.41±0.98	34.00±0.09
Oct	356±1	5.53±1.03	34.05±0.00
Nov	352±0	6.03±0.94	34.05±0.06
Dec	351±0	6.98±0.81	34.00±0.02

Supplementary Table 3. Climatological surface fCO_2, SST and SSS from January to December in the region (48° S, 142.5°–147.5° E) calculated based on the climatological data by Takahashi et al.[7]

month	fCO_2 (μatm)	SST (°C)	SSS
Jan	332±1	11.15±0.29	34.68±0.05
Feb	327±1	11.29±0.29	34.57±0.01
Mar	332±1	11.04±0.26	34.58±0.06
Apr	344±0	10.38±0.16	34.53±0.08
May	338±0	9.96±0.13	34.61±0.10
Jun	347±1	9.44±0.06	34.33±0.06
Jul	354±0	9.13±0.16	34.50±0.12
Aug	364±0	8.93±0.16	34.57±0.04
Sep	360±0	8.92±0.18	34.42±0.05
Oct	350±0	9.07±0.23	34.56±0.01
Nov	338±0	9.65±0.25	34.42±0.12
Dec	334±0	10.51±0.23	34.43±0.01

Supplementary Table 4. Mean January concentrations of phosphate and silicate within the three latitudinal bands of 60°–55° S, 55°–50° S and 50°–45° S between 140.5° and 147.5° E (units of μmol kg^{-1}). These data are from the World Ocean Atlas 2013 (http://www.nodc.noaa.gov/OC5/indprod.html) with a spatial resolution of one degree.

	Phosphate	Silicate
60-55	1.52±0.06	5.32±3.69
55-50	1.24±0.11	2.19±1.19
50-45	0.73±0.19	2.62±1.04

Supplementary Table 5. Observational values of salinity, TA, DIC, [TA−DIC], pH at the regional mean temperature of 7.45 ℃ ($pH_{@7.45}$) and Ω_{arag} at the regional mean temperature of 7.45 ℃ ($\Omega_{arag@7.45}$) at Points S, D and N along transect SR03 during January 1995 and their changes relative to Point N as well as their gradient per salinity change. The position of Points S, D and N also can be found in Fig. 5 in the main text. Units of TA, DIC and [TA-DIC] are μmol kg^{-1}.

	observational values	change relative to Point N	gradient per salinity change
Point S (location: 139.839 °E, 65.585 °S; water depth: 10 dbar)			
salinity	33.41	-0.42	
TA	2258.6	-21.50	50.95
DIC	2129.2	8.00	-18.96
[TA−DIC]	129.4	-29.50	69.91
$pH_{@7.45}$	7.96	-0.07	0.17
$\Omega_{arag@7.45}$	1.5	-0.26	0.62
Point D (location: 139.862 °E, 53.351 °S; water depth: 152 dbar)			
salinity	33.91	0.08	
TA	2287.10	7.00	84.34
DIC	2151.6	30.40	366.27
[TA−DIC]	135.5	-23.40	-281.93
$pH_{@7.45}$	7.97	-0.06	-0.72
$\Omega_{arag@7.45}$	1.51	-0.25	-3.01
Point N (reference point; location: 139.862 °E, 53.351 °S; water depth: 11 dbar)			
salinity	33.83		
TA	2280.1		
DIC	2121.2		
[TA−DIC]	158.9		
$pH_{@7.45}$	8.03		
$\Omega_{arag@7.45}$	1.76		

Supplementary Table 6. Contribution of each process to trend change of SSS (ΔSSS), TA (ΔTA), DIC (ΔDIC) and [TA-DIC] (Δ[TA-DIC]) during the positive SAM trend of 1991-2000 at high-latitudes (60 °–55 ° S). Unit of ΔSSS is PSU yr^{-1} and units of ΔTA, ΔDIC and Δ[TA-DIC] are μmol kg^{-1} yr^{-1}.

Process		ΔSSS	ΔTA	ΔDIC	Δ[TA−DIC]
physical transports	Ekman transport	-0.023	-1.17	0.44	-1.61
	vertical mixing	0.006	0.51	2.20	-1.69
subtotal		-0.017	-0.67	2.63	-3.30
air-sea flux & biology	air-sea CO_2 flux	nc	nc	-0.29 (-0.97)	0.29 (0.97)
	biology	nc	nc	-1.01 (-0.33)	1.01 (0.33)
subtotal		nc	nc	-1.30	1.30

"nc" denotes no change. During calculation of DIC gains by uptake of atmospheric CO_2 ($\Delta DIC_{a\text{-}s}$), t = 30 days and t = 100 days are adopted, respectively (t is the time for CO_2 uptake), which produces two different change rates of $\Delta DIC_{a\text{-}s}$. Blue numbers in the parenthesis show the case of t = 100 days. Details can be found in Methods in the main text.

Supplementary references:

1. Bakker, D. et al. An update to the Surface Ocean CO_2 Atlas (SOCAT version 2). *Earth Syst. Sci. Data* **6**, 69-90 (2014).
2. Schlitzer, R. Ocean Data View. **http://odv.awi.de** (2017).
3. Wanninkhof, R. Relationship between wind speed and gas exchange over the ocean revisited. *Limnology and Oceanography: Methods* **12**, 351-362 (2014).
4. Wanninkhof, R. Relationship between Wind Speed and Gas Exchange Over the Ocean. *J. Geophys. Res.* **97**, 7373-7382 (1992).
5. Atlas, R. et al. A Cross-calibrated, Multiplatform Ocean Surface Wind Velocity Product for Meteorological and Oceanographic Applications. *Bulletin of the American Meteorological Society* **92**, 157-174 (2011).
6. Orsi, A.H., Whitworth III, T. & Nowlin Jr, W.D. On the meridional extent and fronts of the Antarctic Circumpolar Current. *Deep Sea Research Part I: Oceanographic Research Papers* **42**, 641-673 (1995).
7. Takahashi, T. et al. Climatological mean and decadal change in surface ocean pCO_2, and net sea-air CO_2 flux over the global oceans. *Deep Sea Research Part II* **56**, 554-577 (2009).
